# A comprehensive model of tomato fruit ripening regulation by the transcription factors NOR-like1, NAC-NOR, and MADS-RIN

**DOI:** 10.1093/plphys/kiaf291

**Published:** 2025-06-30

**Authors:** Victor Aprilyanto, Xiaowei Wang, Rufang Wang, Stan Kronenberg, Feitse Bos, Cristian Peña-Ponton, Gerco C Angenent, Ruud A de Maagd

**Affiliations:** Laboratory of Molecular Biology, Wageningen University, Wageningen 6700HB, The Netherlands; Laboratory of Molecular Biology, Wageningen University, Wageningen 6700HB, The Netherlands; Institute of Facility Agriculture, Guangdong Academy of Agricultural Sciences, Guangzhou 510640, China; Laboratory of Molecular Biology, Wageningen University, Wageningen 6700HB, The Netherlands; Laboratory of Molecular Biology, Wageningen University, Wageningen 6700HB, The Netherlands; Laboratory of Molecular Biology, Wageningen University, Wageningen 6700HB, The Netherlands; Laboratory of Molecular Biology, Wageningen University, Wageningen 6700HB, The Netherlands; Bioscience, Wageningen Plant Research, Wageningen 6700AA, The Netherlands; Bioscience, Wageningen Plant Research, Wageningen 6700AA, The Netherlands

## Abstract

Tomato (*Solanum lycopersicum*) fruit ripening involves climacteric ethylene production, lycopene accumulation, texture softening, and flavor enhancement, a highly coordinated process accompanied by profound gene expression changes. To construct a comprehensive model of ripening regulation, we studied the effects on ripening phenotypes and underlying gene expression changes in combinations of knockout alleles of *NON-RIPENING-like1* (*NL1*), *NON-RIPENING* (*NAC-NOR*, *NOR*), and *RIPENING INHIBITOR* (*MADS-RIN*). Thus, we demonstrated that the products of the putative paralogous transcription factor genes *NL1* and *NOR* together orchestrate ripening initiation and progression through ethylene production. NL1, or the ethylene production that it induces, together with NOR, stimulates the gene expression of transcription factor MADS-RIN, which then becomes the major driver of all ripening processes studied here. NOR and, particularly, NL1 have relatively minor but discernable and clearly different quantitative contributions to the ripening progression after initiation. Thus, the comprehensive model establishes a hierarchy of gene expression events regulating the start and progression of fruit ripening.

## Teaser

The effects of single and combined mutations reveal the roles of 3 crucial transcription factors and place them in a comprehensive model of tomato fruit ripening regulation.

## Introduction

Ripening is an important part of fleshy fruit development and increases the chances of seed dispersal by frugivores. To achieve this, ripening makes the fruit appear distinct from the other parts of the plant and its environment and palatable to the animals consuming it. A series of physiological changes occur during ripening, including sugar production, flavor and aroma biosynthesis, and softening (Giovannoni 2001; Li et al. 2021). Two types of fruit ripening are broadly distinguished based on the role of the gaseous hormone ethylene. Climacteric ripening is associated with a respiration peak and is usually followed by an ethylene burst, while neither happens in nonclimacteric ripening (Giovannoni 2004). Tomato (*Solanum lycopersicum*) is a climacteric fruit that produces a substantial amount of ethylene during ripening. Ethylene biosynthesis starts from the conversion of *S*-adenosyl-L-methionine (SAM) to 1-aminocyclopropane-1-carboxylic acid (ACC) by ACC SYNTHASE (ACS; Adams and Yang 1979; Boller et al. 1979). Next, ACC is oxidized to ethylene by ACC OXIDASE (ACO; Hamilton et al. 1991). Two ethylene production systems, 1 and 2, respectively, are known to occur in climacteric fruits (McMurchie et al. 1972; Barry et al. 2000). System 1 is active until the onset of ripening, and it is autoinhibitory, meaning that ethylene inhibits its own production, while system 2 takes over at the onset of ripening, is autocatalytic, and responsible for the ripening-related ethylene peak. Despite the presence of multiple *ACS* and *ACO* genes in tomato, only a few change expression during fruit development and ripening (Barry et al. 2000, 1996). Based on these expression changes, *ACS1A* and *ACS6* are active in system 1, while *ACS2* and *ACS4* are active in system 2.

Early discoveries of “ripening transcription factors” (ripening TFs) came from the observation of spontaneous ripening mutants, such as *ripening inhibitor* (*rin*; written as *rin-s* from here onwards), *non-ripening* (*nor*; written as *nor-s* from here onwards), and *Colorless non-ripening* (*Cnr*) (Robinson and Tomes 1968; Tigchelaar et al. 1973; Thompson et al. 1999; Wang et al. 2020). As their names suggest, the fruits from these mutants stay green or pale yellow and firm, whereas a wild-type fruit becomes red and soft. The production of ethylene is also severely diminished in these mutants. The *rin* mutant has a 2.6 kb deletion in the intergenic region between the tandem MADS-box (MCM1, AGAMOUS, DEFICIENS, and SRF) genes *RIPENING INHIBITOR* (*MADS-RIN*) and *MACROCALYX* (*MADS-MC*), which results in a *rin-mc* fusion gene that was thought to abolish the *MADS-RIN* function (Vrebalov et al. 2002). However, a re-evaluation via CRISPR/Cas9 knockout of both *MADS-RIN* (abbreviated as *RIN* from here onwards) and the mutant *rin* allele showed a milder ripening phenotype than the *rin-s* mutant. These observations led to the conclusion that *rin-mc* is a gain-of-function mutation that combines the DNA binding by *RIN* and the negative transcription regulation by *MADS-MC* (Ito et al. 2017, 2015; Li et al. 2020).

Like *rin-s*, the fruit of *nor-s* mutant produces little ethylene and stays green and firm. The *nor-s* allele has a 2 bp deletion in the third exon of the NAC (NAM, ATAF, and CUC) gene *NAC-NOR* (*NON-RIPENING*; abbreviated as *NOR* from here onwards), which produces a C-terminally truncated protein, making it initially also regarded to be a loss-of-function mutation (Giovannoni et al. 2004). Using mutagenesis by CRISPR/Cas9, it was later shown that a true *nor* knockout mutant (*nor-cr*) fruit had a milder ripening phenotype (Wang et al. 2019b; Gao et al. 2020). Moreover, the knockout of the *nor-s* allele (*nor-scr* mutant) also partially restored ripening similar to the level of *nor-cr*, demonstrating that the truncated protein produced by *nor-s* is responsible for the nonripening phenotype and represents a (trans) dominant-negative mutation (Wang et al. 2019b). In correspondence with the phenotype, *nor-cr* fruit produces less ethylene and lycopene during ripening due, at least in part, to reduced expression of system 2 ethylene biosynthesis genes (e.g. *ACS2* and *ACO3*) and carotenoid biosynthesis genes *GERANYLGERANYLDIPHOSPHATE SYNTHASE 2* (*GGPPS2*) and *PHYTOENE SYNTHASE 1* (*PSY1*), respectively (Wang et al. 2019b; Gao et al. 2020). Moreover, the fruit of *nor-cr* was significantly firmer, which is associated with a reduced expression of cell wall metabolism genes, e.g. *POLYGALACTURONIDASE 2a* (*PG2a*) and *PECTATE LYASE* (*PL*) (Gao et al. 2020). The reduced expression of these ripening-related genes in the *nor* knockout suggests that NOR might directly regulate them. Indeed, some of these genes were shown to be directly bound and activated by NOR due to the presence of NAC TF binding sites in the promoter region (Gao et al. 2020).

NOR-like1 (abbreviated as NL1 from here onwards), previously also named NAC3 (Jing et al. 2018) or SNAC4 (Kou et al. 2016), is another NAC TF that has been shown to regulate tomato ripening (Gao et al. 2018). In addition to its highly similar protein sequence, NL1 shares functions similar to NOR in ripening regulation. The silencing or knockout of *NL1* reduced the ripening ethylene production, color development, and fruit softening. Likewise, the expression of genes related to those traits was downregulated (Gao et al. 2018; Kou et al. 2018, 2016). Despite the similarity to *NOR*, *NL1* has additional regulatory roles in seed and preripening fruit development (Han et al. 2014; Guan et al. 2023; Peng et al. 2023).

Due to the similar ripening phenotypes caused by the knockout of each gene, *NOR* and *NL1* are likely to coregulate ripening together, particularly the system 2 ethylene biosynthesis (Gao et al. 2018, 2020; Wang et al. 2019b). However, since there is currently little data on the effect of multiple TF knockouts on ripening, it is unknown whether these TFs regulate ripening independently, redundantly, additively, or synergistically. This study aims to determine the mode of ripening regulation by *NOR* and *NL1*, as well as their mutual interaction and that with the ripening regulator *RIN*. It addresses this question by comparing the phenotypes and ripening-related gene expression of combinations of *nor* and *nl1* knockout alleles and the combination of *rin* and *nor* mutations.

## Results

### Generation of *NAC-NOR* and *NOR-like1* knockout mutants

Using CRISPR/Cas12a, we produced knockout mutations in *NL1* using crRNAs targeting the first exon. We obtained biallelic (Δ4 bp/Δ6 bp) and heterozygous (Δ4 bp/+) T_0_ mutants. In the next generation (T_1_), we subsequently segregated transgene-free homozygous mutants containing the 2 distinct 4 bp deletion mutations. Both mutations cause a translation frameshift, after amino acids 29 and 28, respectively, and truncated proteins (Supplementary Fig. S1A). Next, we generated allelic combinations of *nor nl1* double mutants by crossing *nor-cr1* obtained from the previous study (Supplementary Fig. S1B; Wang et al. 2019b) with *nl1-cr1* from this study (named as *nor-cr1 nl1-cr1*). The *nor-cr1 nl1-cr1* double homozygous mutants failed to set fruits or produced abnormally small seedless fruits, as also seen by others (Li et al. 2025). Manual pollination with wild-type pollen resulted in normally developing, seeded fruits, indicating that the combined *nor* and *nl1* mutations led to a male fertility defect. As reported in the paper by Li et al., this was due to pollen wall collapse and severely reduced viability and germination. The same paper reports that the single mutations had no such effects, which we confirmed. The fruits derived from manual pollination of *nor-cr1 nl1-cr1* with wild-type pollen were used for further phenotyping.

### Color development is inhibited in the *nor* knockout and is lacking in the double knockout mutant

**Figure 1.**
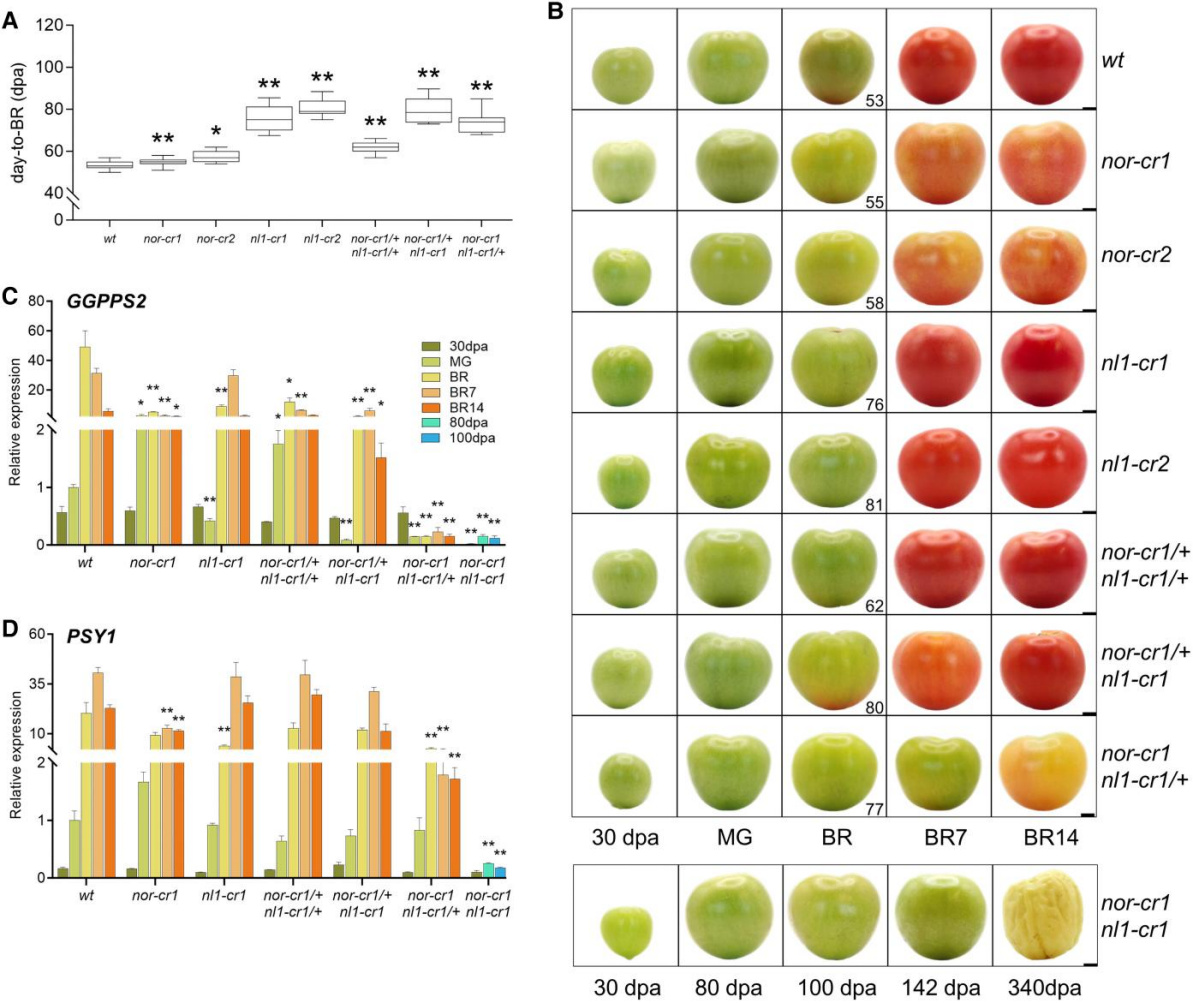
Fruit ripening phenotypes. **A)** Days-to-breaker of the wild type and mutants. The *nor-cr1 nl1-cr1* double homozygous mutant was not included since the ripening initiation of this mutant could not be established. At least 25 fruits of each genotype were used in the comparison. For the box plot in panel (**A**), the center line represents the median; box limits indicate the upper and lower quartiles (Q3 and Q1, respectively); and whiskers extend to 1.5 times the interquartile range (IQR) from the quartiles. The asterisks indicate significant differences using a 2-tailed Student's *t*-test at *P* < 0.05 (*) and *P* < 0.01 (**) between each mutant and the wild type. **B)** Fruit appearance from 30 d postanthesis (30 dpa) to breaker + 14 d (BR14) from the wild type and the *nor-cr* and *nl1-cr* single and double mutants. For the *nor-cr1 nl1-cr1* double homozygous mutant, fruits were harvested at 30, 80, 100 dpa, while the fruits of 142 and 340 dpa were harvested at 100 dpa. Bar = 1 cm. Gene expression by RT-qPCR for carotenoid biosynthesis pathway genes **C)**  *GGPPS2* and **D)**  *PSY1*. For the bar graphs in panels (**C**) and (**D**), error bars represent the standard error (SE) of the mean, with sample sizes of *n* = 3 biological replicates for each treatment. Asterisks indicate significant differences using a 2-tailed Student's *t*-test at *P* < 0.05 (*) and *P* < 0.01 (**) between each mutant and wild type at the same developmental stages, except for the *nor-cr1 nl1-cr1* double homozygous mutant, where both 80 and 100 dpa stages were compared to wild type MG. Abbreviations: 30 dpa, 30 d postanthesis; MG, mature green; BR, breaker; BR7, breaker + 7 d; BR14, breaker + 14 d.

In this study, we defined ripening initiation as the transition from the mature green (MG) to breaker stages that is indicated by an observable color change occurring at the pericarp stylar end. We then defined ripening progression as the phenotypic changes after ripening initiation. This distinction is important to specifically address the effect of *nor* and *nl1* knockout mutations on ripening aspects. First, we compared the ripening initiation and progression between the wild type, *nor-cr* and *nl1-cr* single, and all allelic combinations of *nor* and *nl1* double knockout mutants. The wild-type fruits reached the breaker stage around 53 d postanthesis (dpa), and from this point, the fruit became fully red over the next 7 d (Fig. 1, A and B). The change of color in ripening progression matches well with the changes in fruit pigments as measured by remittance spectroscopy, where chlorophyll content decreased, and lycopene content increased (Supplementary Fig. S2). Similar to what was reported earlier (Wang et al. 2019b), both *nor-cr* mutants were slightly delayed initiating ripening, but slowly progressed through ripening (Fig. 1, A and B). This was supported by a slower rate of both chlorophyll degradation and lycopene accumulation, resulting in an orange-colored fruit at 7 and 14 d postbreaker stages (Supplementary Fig. S2). In contrast, both *nl1-cr* mutants had a much longer delay of ripening initiation (Fig. 1A), as observed previously (Gao et al. 2018). During the ripening (from the breaker stage onwards), we observed no visible color difference to the wild type (Fig. 1B, Supplementary Fig. S2).

Combinations of knockout alleles from the 2 genes revealed an allele dosage effect on ripening initiation and progression. The *nor-cr1/+ nl1-cr1/+* double heterozygous mutant showed a delayed ripening initiation. Although visually similar, the *nor-cr1/+ nl1-cr1/+* mutant accumulated significantly less lycopene at breaker + 7 d (Supplementary Fig. S2B). Leaving just a single functional *NL1* copy (as in *nor-cr1 nl1-cr1/+*) resulted in yellow-colored fruits, which was a more severe effect compared to leaving a single functional *NOR* copy (as in *nor-cr1/+ nl1-cr1*) that still turned red albeit delayed (Fig. 1B). This was further confirmed by spectroscopy which showed higher lycopene accumulation for the latter mutant. Most strikingly, we did not observe any color change up to 142 dpa, and only general senescence at 340 dpa when all copies of *NOR* and *NL1* were knocked out (as in *nor-cr1 nor-like1-cr1*) (Fig. 1B). Daily measurements on the fruit of this double homozygous mutant from 80 dpa to 7 d afterward showed no chlorophyll degradation or lycopene accumulation, further supporting that this mutant did not ripen at all. As there was neither ripening initiation or progression observed in this double mutant, we chose the fruits of 80 and 100 dpa, far beyond the time to breaker of any of the other mutants, for comparison with the MG stages (defined as 5 d prior to the breaker stage) of the wild type and other mutants.

Since ripening in tomato starts from the inner tissues (Brecht 1987), we also compared the color difference between those (jelly, columella, and placenta) and pericarp at the MG and breaker stages. There was no development of red color in either tissue at the MG stage, while at the breaker stage, the inner tissue generally showed a more intense red color than the pericarp (Supplementary Fig. S3). A similar pattern was observed in the *nor-cr* mutants, although the color of the inner tissues and pericarp was lighter than that of the wild type. Interestingly, the breaker stage of *nl1-cr* mutants had their pericarp developing a more intense red color than the inner tissue, suggesting that the pericarp ripened earlier. In the double mutants, the allelic combinations followed the patterns of the respective single mutants. The *nor-cr1/+ nl1-cr1* mutant exhibited color change in the pericarp earlier than in the inner tissue, whereas the *nor-cr1 nl1-cr1/+* mutant barely developed red color in either the pericarp or inner tissue upon ripening. Finally, the *nor-cr1 nl1-cr1* double homozygous mutant showed no color change in either tissue.

In relation to lycopene production, we compared the expression of the genes upstream in the biosynthetic pathway, *GGPPS2*, and *PSY1*, in the pericarp tissue between the wild type and mutants. In the wild type, both *GGPPS2* and *PSY1* expression increased during ripening, with peak expression at the breaker and 7 d afterward, respectively (Fig. 1, C and D). The knockout of *NOR* lowered the expression of both during ripening, explaining the orange phenotype of the mutant. This was in contrast with the knockout of *NL1*, which lowered *GGPPS2* and *PSY1* expression only at the MG and breaker stages but not at the later stages (Fig. 1, C and D). This matched the higher final lycopene content in the *nl1* compared to *nor* knockout mutants. The combined knockout alleles of *nor* and *nl1* showed an allele dosage effect on the expression of *GGPPS2* and *PSY1*, with a more profound decrease in the mutant with decreasing functional *NOR* compared to decreasing functional *NL1*. This correlated well with the observed fruit appearance and measurement of color change during ripening. Lastly, there was no increase in *GGPPS2* and *PSY1* expression in the *nor-cr1 nl1-cr1* double homozygous mutant at 80 or 100 dpa, matching the absence of ripening color in this mutant (Fig. 1, C and D). To summarize, both *NOR* and *NL1* regulate the ripening process, with *NL1* regulating the ripening initiation and *NOR* and *NL1* together, with a bigger role for *NOR*, regulating ripening progression. The wild-type alleles of both genes act additively during ripening, as revealed by the allele dosage effect, and without an active allele of either of the two, ripening is inhibited altogether.

### *NAC-NOR* and *NL1* regulate ethylene production additively

**Figure 2.**
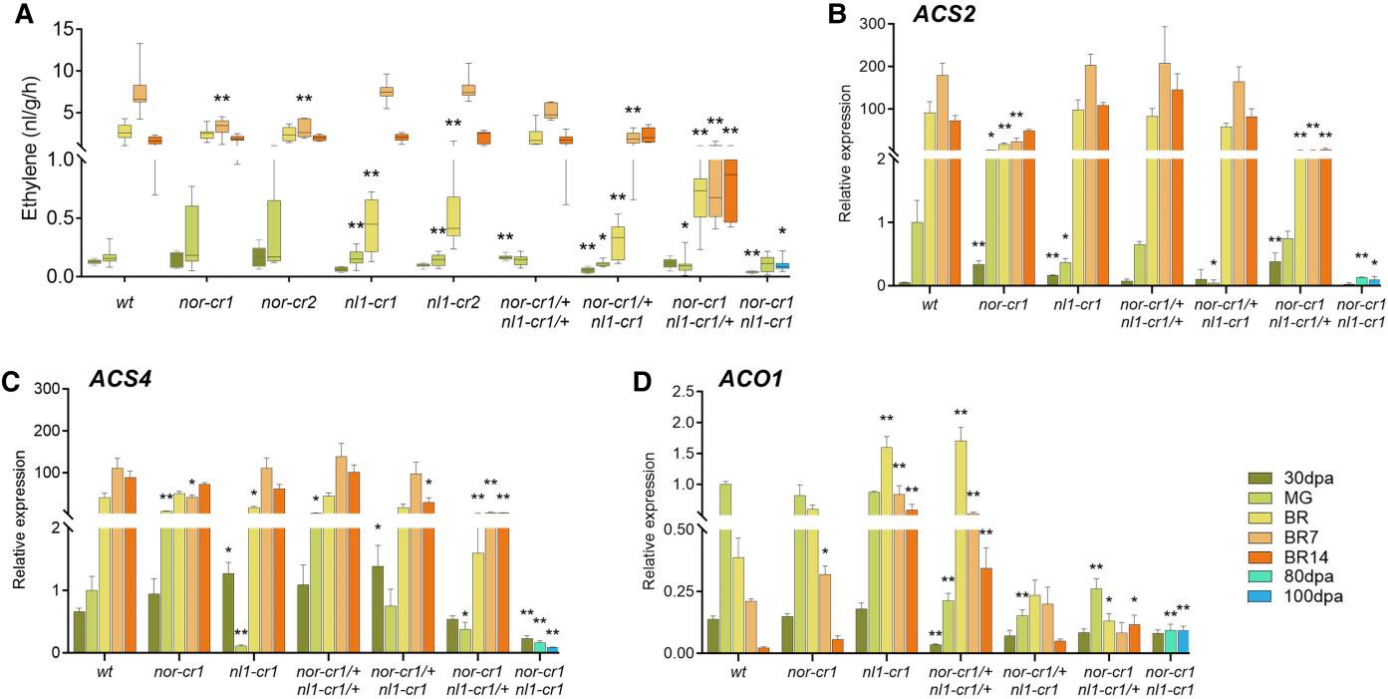
Ethylene production and the expression of system 2 ethylene biosynthesis genes in wild type and mutants at 5 developmental and ripening stages. **A)** Ethylene production. For the *nor-cr1 nl1-cr1* double homozygous mutant, ethylene production was measured at 30, 80, and 100 dpa. At least 5 fruits per stage from each genotype were used for the measurements. For the box plot in panel (**A**), the center line represents the median; box limits indicate the upper and lower quartiles (Q3 and Q1, respectively); and whiskers extend to 1.5 times the interquartile range (IQR) from the quartiles. The expression of system 2 ethylene biosynthesis genes comprises **B)**  *ACS2*, **C)**  *ACS4*, and **D)**  *ACO1*. For the bar graphs in panels (**B**) to (**D**), error bars represent the standard error (SE) of the mean, with sample sizes of *n* = 3 biological replicates for each treatment. Asterisks indicate significant differences using a 2-tailed Student's *t*-test at *P* < 0.05 (*) and *P* < 0.01 (**) between each mutant and the wild type at the same developmental stages, except for the *nor-cr1 nl1-cr1* double homozygous mutant, where both 80 and 100 dpa stages were compared to MG wild type.

Ethylene production is a prominent feature of climacteric ripening in tomato. In the wild type, fruit ethylene production was negligible at 30 dpa and at the MG stage but rose significantly toward the breaker stage and the next 7 d (Fig. 2A). The overall climacteric rise of ethylene production was reduced in *nor-cr* and *nl1-cr* single homozygous knockout lines, confirming their previously reported regulatory roles in ethylene production (Gao et al. 2018, 2020; Wang et al. 2019b). Despite the similarity, the dynamics of ethylene production differed between the mutants. In the *nor-cr* mutants, while not significantly different up to the breaker stage, the peak production at the breaker + 7 d stage in this mutant was much lower than the wild type. This was in contrast with *nl1-cr* mutant fruits which produced significantly less ethylene at the breaker stage, but rose to wild-type levels afterwards (Fig. 2A). In parallel with color development, we then observed a gradual decrease in peak ethylene production when more alleles of *NOR* and *NL1* were knocked out. The mutant with 1 functional *NOR* allele (*nor-cr1/+ nl1-cr1*) produced more ethylene than that having 1 functional *NL1* allele (*nor-cr1 nl1-cr1/+*). Like the single *nl1-cr* mutants, the *nor-cr1/+ nl1-cr1* fruit also produced much less ethylene at the breaker stage. This underlines the similar yet not identical role of the 2 genes in regulating ethylene production, where *NL1* impacts early ripening (the breaker stage) production and *NOR* regulates later ethylene production, with the latter having the major role. A residual role of *NL1* in late ethylene production became apparent only in the *nor-cr1 nl1-cr1/+* genotype, producing less ethylene than the *nor-cr1* single mutant. Lastly, the absence of ethylene production in the double homozygous mutant (*nor-cr1 nl1-cr1*) suggests that ripening ethylene production is triggered by these 2 genes in an additive manner.

To understand the dynamic of ethylene biosynthesis regulation at the molecular level, we compared the expression of system 2 ethylene biosynthesis genes between genotypes. The expression of *ACS2* and *ACS4* in the wild type was correlated with ethylene production, with expression rising more than 100-fold and 50-fold for *ACS*2 and *ACS4*, respectively, at breaker and peaking 7 d later (Fig. 2, B and C). Overall, *ACS2* expression was more severely impacted by the mutations than *ACS4* expression was. In *nor-cr1*, *ACS2* expression was significantly lower in all ripening stages (from the breaker to breaker + 14 d stages), in contrast to its expression in *nl1-cr1*, which was lower only at the MG stage. This pattern, albeit weaker, is also observed for *ACS4*, where *nor-cr1* had a reduced expression at the breaker + 7 d stage. While the *nor-cr1/+ nl1-cr1/+* double heterozygous mutant showed no significantly lower *ACS2* and *ACS4* expression (Fig. 2, B and C), further removal of an additional *NOR* allele had a bigger impact than that of an extra *NL1* allele, which is consistent with the ethylene production (Fig. 2A). The contribution of *NL1* in regulating *ACS2* and *ACS4* expression can be seen when comparing expression in the mutants with nonfunctional *NOR* alleles. Here, *nor-cr1 nl1-cr1/+* had lower expression of *ACS2* and *ACS4* than the nor-cr1 mutants (Supplementary Table S1), showing that although small, NL1 contributes to the regulation of ethylene biosynthesis genes. Finally, both *ACS2* and *ACS4* were expressed at very low levels in the double homozygous mutant (Fig. 2, B and C). This suggests that the overall expression of *ACS2* and *ACS4* and resulting ethylene production (Fig. 2A) is controlled by *NL1* at the onset of ripening and then by both *NL1* and *NOR* during the progression of ripening.

Additionally, *ACO1* expression was also affected in the mutants. In the wild type, *ACO1* expression peaked at the MG stage and went down at later stages of ripening. A similar pattern was also shown in the *nor-cr1* mutant, although with a slower rate of decrease, which resulted in a higher expression than the wild type at the breaker + 14 d stage (Fig. 2D). Despite a similar decreasing expression trend in the *nl1-cr1* mutant, the expression levels for each stage during ripening were significantly higher than the wild type, suggesting that the knockout shifts the *ACO1* expression peak, reflecting the ripening delay in the mutants. When additional *NOR* and *NL1* functional alleles were lost, *ACO1* expression was further decreased. This indicates that *NOR* and *NL1* also control ethylene production through *ACO1* expression, although *ACS2* and *ACS4* expression correlates better with actual ethylene production.

### Substantial ripening restoration by external ethylene occurs only when a functional *NOR* allele is present

**Figure 3.**
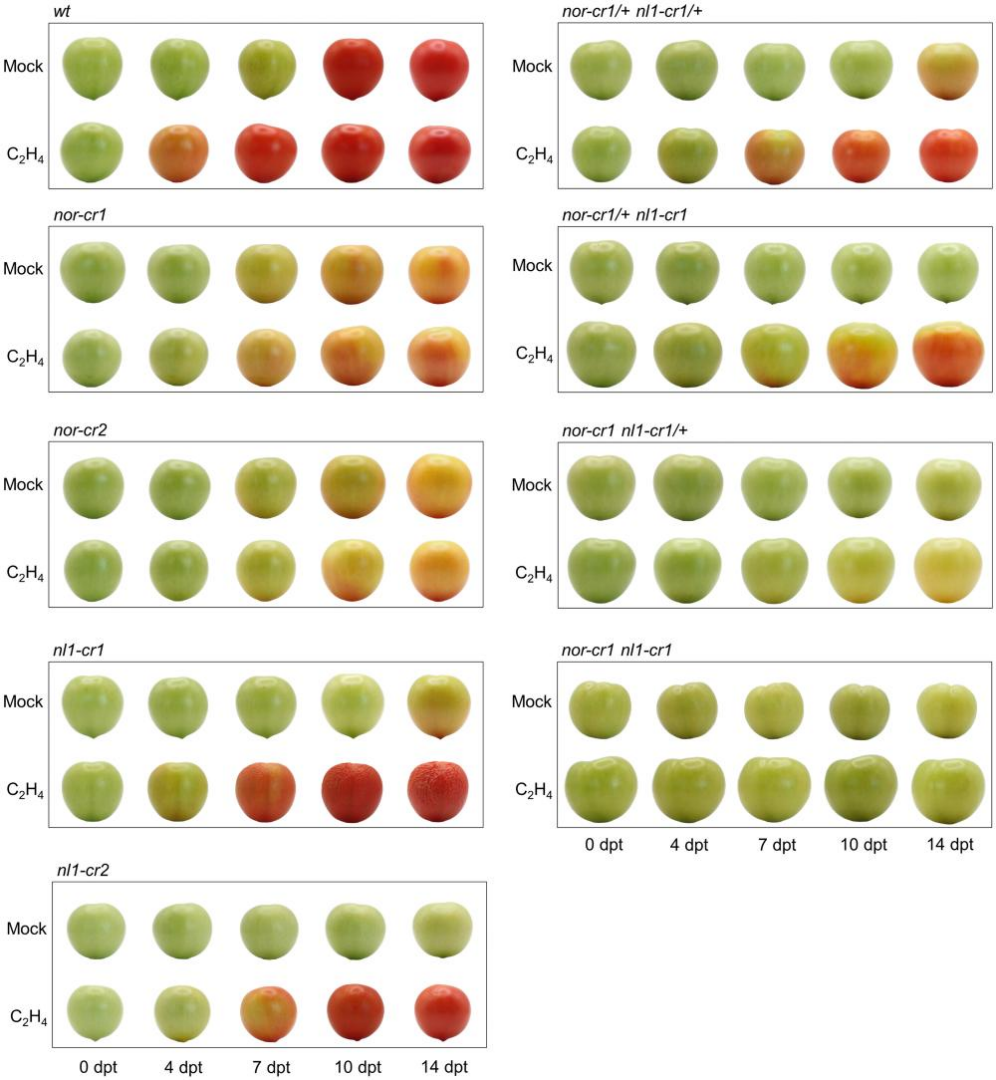
Comparison between the wild type and the single and double mutants of *nor* and *nl1* fruits treated with water (mock; upper rows) and ethephon (lower row) from 0 to 14 dpt. Images were digitally extracted for comparison.

The observation of reduced ethylene production and its biosynthetic gene expression before and during ripening in the *nor* and *nl1* mutants suggests that ethylene production drives both ripening initiation and progression. Since the lack of ethylene precludes both the initiation and progression of ripening, we hypothesized that supplying external ethylene would restore these phenotypes in at least some of the mutants. To test this, we treated the wild type and mutant fruits with ethephon solution and observed the fruit color development over 14 d posttreatment (dpt). Most of the ethephon-treated fruits initiated ripening earlier than the mock-treated fruits, although ripening progression varied between mutants containing *nor* and *nl1* knockout alleles (Fig. 3). In the *nor-cr* mutants, both ripening initiation and progression were visually similar between the mock and ethephon-treated fruits, confirming that a functional *NOR* and sufficient ethylene are required. This contrasted with the *nl1-cr* mutants, where ethephon treatment initiated earlier ripening and accelerated its progression (breaker at 4 dpt) compared to the mock treatment (breaker at 14 dpt), showing that a sufficient level of initial ethylene is required to initiate ripening and that this is, at least mainly, controlled by *NL1* (Fig. 3).

This was further supported by earlier ripening initiation in the fruits of both *nor-cr1/+ nl1-cr1* and *nor-cr1 nl1-cr1/+* double mutants in ethephon treatment. However, only the former was able to progress through ripening similar to normal. Finally, the ethephon treatment on *nor-cr1 nl1-cr1* fruit did not change the fruit color even after 14 dpt, indicating that this mutant did not ripen even with externally supplied ethylene (Fig. 3). This suggests that ethylene production is controlled by *NL1* as well as by *NOR* activity and that both are required for normal ripening. Taken together, we showed that a sufficient level of initial ethylene is required to initiate ripening and that NL1 controls this. After that, the continuation of ethylene production in ripening progression requires both *NOR* and *NL1*.

### Fruit firmness is differentially regulated by *NAC-NOR* and *NL1*

Fruit firmness decreases during ripening. The on-the-vine firmness of the wild-type fruit peaked at the MG stage and subsequently decreased during ripening (Supplementary Fig. S4A). The *nor-cr* mutant fruits were firm at the MG stage, but softened slower than the wild type during ripening, resulting in firmer fruits at the breaker + 14 d stage. In contrast, the *nl1-cr* mutants had much firmer fruits at the MG stage but softened faster during ripening, resulting in slightly firmer fruits at the breaker + 14 d stage. Combining *nor* and *nl1* knockout alleles produced an aggregate of what was observed in the single knockout mutants. The *nor-cr1/+ nl1-cr1/+* double heterozygous had a higher firmness at MG and softened at a slightly slower rate compared to the wild type. From this point, further removal of the *NL1* allele resulted in fruit with higher firmness at MG but softening at a rate similar to the wild type, while removal of the *NOR* allele gave a fruit with normal preripening firmness but softened slower. Lastly, the fruit of the *nor-cr1 nl1-cr1* double homozygous mutant had a significantly higher preripening firmness that did not decrease throughout 100 dpa. Taken together, it can be concluded that fruit softening, controlled by *NL1*, occurs prior to ripening initiation and that it is accelerated under combined control by *NL1* and *NOR* during ripening progression.

We studied the expression of *PG2a* and *PL* which are associated with loss of viscosity and fruit softening (Wang et al. 2019a). *PG2a* is one of the most strongly induced genes during ripening, and its expression in the wild type increased to more than 1000-fold at the breaker + 7 d stage (Supplementary Fig. S4B). *PL* expression increased 50-fold, peaking at the same stage (Supplementary Fig. S4C). The expression of both genes in *nor-cr1*, but not for the *nl1-cr1*, was lower at the breaker + 7 d stage, indicating that *NOR* controls more of their expression than *NL1* does. Further depletion of functional *NOR* alleles resulted in lower expression of *PG2a* and *PL* than that of an *NL1* allele. Finally, no significant expression increase occurred in the double homozygous (*nor-cr1 nl1-cr1*) mutant at 80 and 100 dpa. By comparing the gene expression with the fruit firmness phenotype, we showed that the expression change of *PG2a* and *PL* was associated negatively with the fruit firmness during ripening but not before it. Although the higher *PG2a* and particularly *PL* expression could explain the lower fruit firmness in the *nor-cr1* mutant at the MG stage, it could not satisfactorily explain the much higher fruit firmness in the mutants containing the *nl1-cr1* mutation. This indicates that the change in fruit firmness, due to the activities of cell wall metabolism genes other than *PG2a* and *PL*, has occurred in the early fruit development, and NL1 regulates it.

### *NOR* and *NL1* additively regulate the expression of ripening TFs

**Figure 4.**
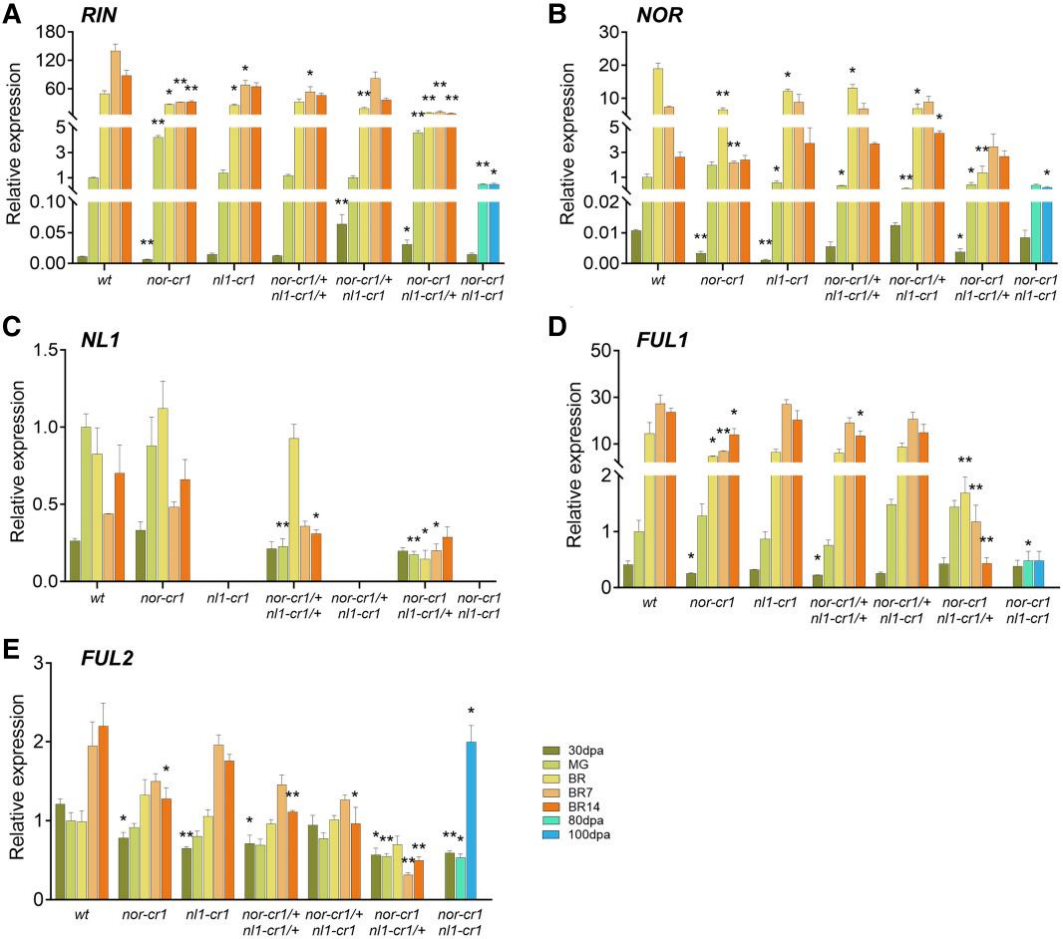
Expression of ripening regulating TF genes **A)**  *RIN*, **B)**  *NOR*, **C)**  *NL1*, **D)**  *FUL1*, and **E)** FUL2. For the bar graphs in all panels, error bars represent the standard error (SE) of the mean, with sample sizes of *n* = 3 biological replicates for each treatment. Asterisks indicate significant differences using a 2-tailed Student's *t*-test at *P* < 0.05 (*) and *P* < 0.01 (**) between each mutant and wild type at the same developmental stages, except for the *nor-cr1 nl1-cr1* double homozygous mutant, where 80 and 100 dpa stages were compared to wild type MG. No statistical test was conducted for *NL1* expression in the lines with complete *nl1-cr1* knockout due to its very low expression.

Considering that the knockouts of *NOR*, *NL1*, or both downregulate many ripening-related genes, we hypothesized that they may also affect the expression of other ripening TFs. To investigate this, we compared the expression of *RIN*, *NOR*, *NL1*, *FUL1*, and *FUL2* in the single and double mutants of *nor* and *nl1*. *RIN* plays a significant role in regulating ripening, and its knockout causes a marked decrease in ethylene production and other ripening aspects (Ito et al. 2017; Li et al. 2020). It is also the most upregulated TF during ripening as far as tested here, more so than *FUL1* and *NOR* and much more so than *FUL2* and *NL1* (Fig. 4A). Our analysis revealed that *RIN* expression was downregulated in both *nor* and *nl1* single mutants, with the downregulation being more pronounced in the *nor* than in the *nl1* knockout. In the allelic combinations of the double mutants, *RIN* expression was only reduced further in the complete knockout of *NOR*. Furthermore, there was no increase in *RIN* expression in the *nor nl1* double homozygous mutant at 80 and 100 dpa, indicating that *RIN* expression is influenced by NOR and, to a lesser extent, by NL1.

We also investigated the effect of the knockouts on the expression of *NOR*. Like *RIN*, *NOR* expression was downregulated in both *nor* and *nl1* single mutants, with the downregulation being more pronounced in *nor* than in *nl1* (Fig. 4B). Interestingly, at the MG stage, *NOR* expression was significantly lower only in mutants with partial or complete *NL1* knockout, suggesting that *NL1 regulates NOR*. In the *nor nl1* double homozygous mutant, *NOR* expression was further downregulated, with no significant increase observed at 80 and 100 dpa. These findings suggest that both *NOR* and *NL1* additively regulate *NOR* transcription levels.

Despite its high sequence similarity with *NOR*, the expression pattern of *NL1* differed significantly from that of *NOR* (Fig. 4C). Interestingly, there were no significant changes in *NL1* expression in the *nor* single mutant, suggesting that *NOR* does not regulate *NL1* expression. However, *NL1* transcripts were not detectable in mutants with a complete *nl1* knockout, likely due to transcript degradation via the nonsense-mediated mRNA decay (NMD) pathway (Lykke-Andersen and Jensen 2015). This presumed degradation of *NL1* transcripts prevented us from assessing the effect of the *nl1* knockout on its own expression.

Both FUL1 and FUL2 are TFs belonging to the MADS family, and during ripening, they interact with RIN to regulate downstream genes, including in the ethylene biosynthesis pathway (Bemer et al. 2012; Wang et al. 2019b). Despite their high similarity in protein sequence, the expression patterns of both *FULs* differed in the *nor* and *nl1* mutants. The expression of *FUL1* was significantly downregulated in mutants containing a homozygous *nor* knockout (Fig. 4, D and E). Furthermore, *FUL1* expression decreased further with the additional knockout of *NL1*, eventually resulting in no expression increase in the *nor nl1* double homozygous mutant. These findings indicate that *NOR* and *NL1* regulate *FUL1* expression either directly or indirectly.

### RNA-seq analysis of gene expression prior to ripening

**Figure 5.**
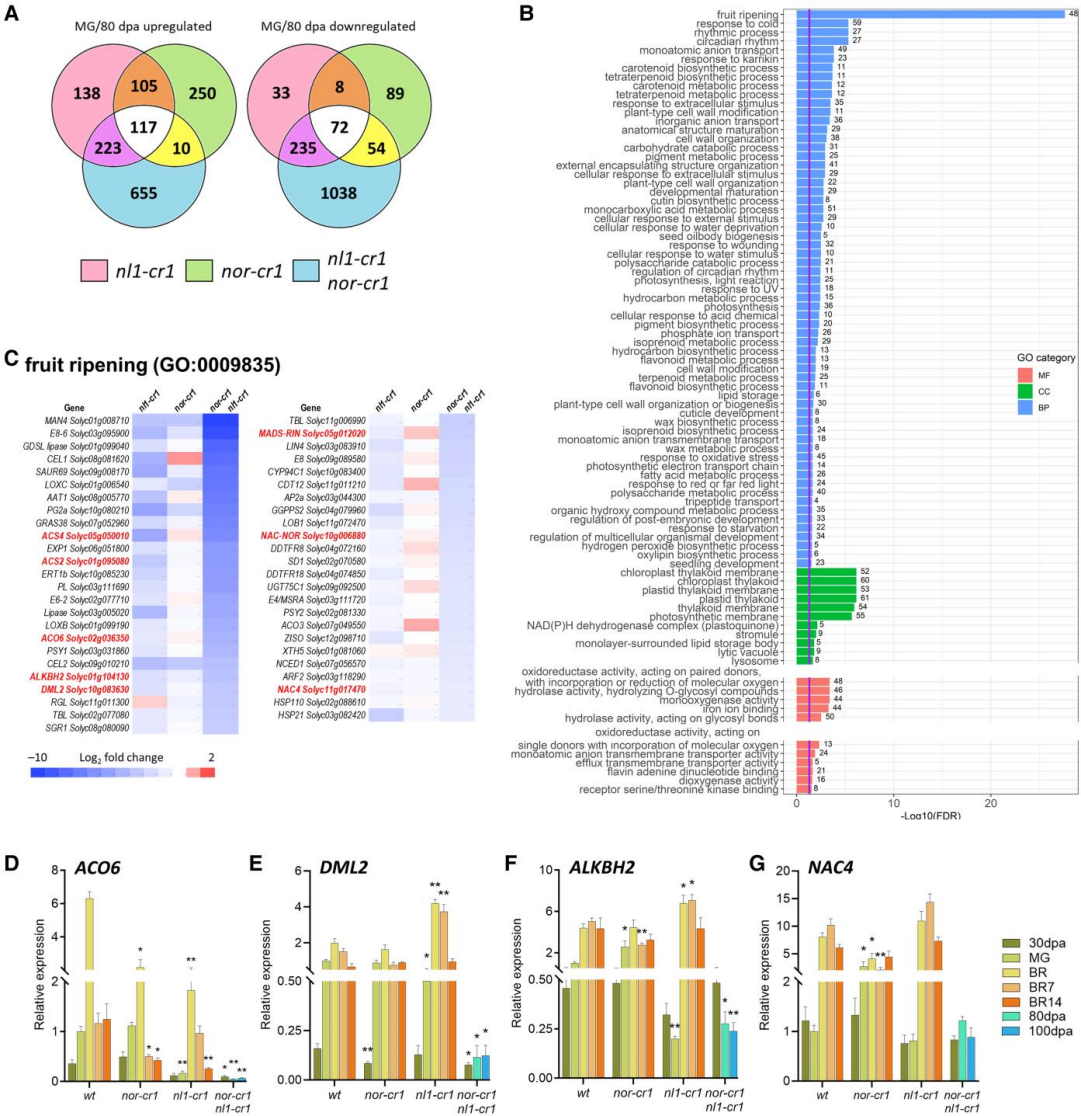
Most fruit ripening-related genes were downregulated in the *nor-cr1 nl1-cr1* mutant at 80 dpa. **A)** Venn diagrams showing the up- and downregulated DEG overlaps between *nl1-cr1*, *nor-cr1*, and *nor-cr1 nl1-cr1* mutants at the MG stage. For *nor-cr1 nl1-cr1*, the DEGs from 80 dpa were used for comparison. **B)** GO enrichment on the downregulated genes of *nor-cr1 nl1-cr1*. **C)** Heatmap of the genes comprising the “fruit ripening” GO term (GO:0009835) compared between *nl1-cr1*, *nor-cr1*, and *nor-cr1 nl1-cr1* mutants. RT-qPCR results of the several ripening genes compared between the wild type and mutants, comprising **D)**  *ACO6*, **E)**  *DML2*, **F)**  *ALKBH2*, and **G)**  *NAC4*. For the bar graphs in panels (**D**) to (**G**), error bars represent the standard error (SE) of the mean, with sample sizes of *n* = 3 biological replicates for each treatment. Asterisks indicate statistical significance based on a 2-tailed Student's *t*-test at *P* < 0.05 (*) and *P* < 0.01 (**) between mutants to wild type at the same developmental stage, except for the *nor-cr1 nl1-cr1* double homozygous mutant, where the 80 and 100 dpa stages were compared to wild type MG.

The comparison of phenotypes and expression of several ripening-related genes among the mutants further supports the hypothesis that *NL1* regulates ripening initiation and both *NL1* and *NOR* additively regulate ripening progression. However, the relative contribution of both *NL1* and *NOR* in these processes required further investigation. To identify which ripening genes are regulated by both *NL1* and *NOR* and why knocking out both *NOR* and *NL1* abolishes ripening completely, we did an RNA-seq of the pericarp tissue at the MG stage. In the case of *nor-cr1 nl1-cr1* mutant, we used 80 dpa fruit as the stage comparable to the MG stage since it showed no signs of ripening. Pericarp tissue from at least 3 different fruits at the defined stages was used for each biological replicate. Detection of the differentially expressed genes (DEGs) was based on a cutoff threshold of |log2fold| > 1 and FDR < 0.05 (Supplementary Table S3). Venn diagrams were made to compare and assess overlap between mutants of numbers of up- or downregulated DEGs (Fig. 5A). At MG, the *nl1-cr1* had 583 up- and 348 downregulated DEGs compared to the wild type, while the *nor-cr1* had 482 up- and 223 downregulated DEGs. These numbers varied significantly compared to the *nor-cr1 nl1-cr1* double mutant, which, at 80 dpa, had 1,005 up- and 1,399 downregulated DEGs. We observed that more of these DEGs were shared between the double mutant and *nl1-cr1* compared to the *nor-cr1*, indicating that the *NL1* knockout induced more profound transcriptomic changes than the *NOR* knockout at this stage.

A GO enrichment from the downregulated DEGs showed that the *nor-cr1 nl1-cr1* double homozygous mutant had an enrichment of “fruit ripening” GO term (GO:0009835), showing that the knockout of both *NL1* and *NOR* downregulates the expression of most ripening-related genes (Fig. 5B; Supplementary Table S4). Interestingly, this GO term enrichment was shared only with the *nl1-cr1* mutant (Supplementary Fig. S5; Supplementary Table S5), suggesting that knocking out *NL1* is necessary to downregulate those genes and leading to the ripening initiation delay observed in the mutant. The absence of the “fruit ripening” GO term in the *nor-cr1* mutant (Supplementary Fig. S6; Supplementary Table S6) indicates that the effect of *nor-cr1* mutation only minimally affected the expression of ripening genes before ripening is initiated.

Gene comparison within the “fruit ripening” GO term between the *nl1-cr1* and *nor-cr1 nl1-cr1* mutants revealed that most genes associated with the former were subsets of those found in the latter (Fig. 5C). This included some of the most common ripening-related genes responsible for: ethylene biosynthesis (*ACS2*, *ACS4*, *ACO6*), color development (*GGPPS2*, *PSY1*, *SGR1*, *ZISO*), cell wall metabolism (*PG2a*, *PL*, *CEL1*, *CEL2*), and ripening-related demethylases *DEMETER-LIKE 2* (*DML2*) and *AlkB HOMOLOG 2* (*ALKBH2*). While the expression of some of these genes was downregulated in the *nl1-cr1* mutant at the MG stage (Figs. 1, C, D, 2, B to D and 4B), other genes like *ACO6*, *DML2*, and *ALKBH2* also followed the same trend (Fig. 5, D to F).

In addition to the downregulated ripening-related genes in the *nl1-cr1* mutant, more genes were downregulated in the *nor-cr1 nl1-cr1* double homozygous mutant. Among the others are the ripening TFs, such as *NOR*, *RIN*, and *NAC4*, which are known to regulate ethylene biosynthesis during ripening (Fig. 5C) (Zhu et al. 2014; Ito et al. 2017; Wang et al. 2019b; Gao et al. 2020, 2021; Li et al. 2020). As shown in Fig. 4, A and B, the expression of both *RIN* and *NOR*, in the *nor-cr1 nl1-cr1* double mutant, was very low at both 80 and 100 dpa. Since both TFs regulate the expression of system 2 ethylene biosynthesis genes, their downregulation in the double mutant explains the low *ACS2* and *ACS4* expression and, subsequently, the absence of ripening. Surprisingly, the expression of *RIN* was significantly higher in the *nor-cr1* mutant at the MG stage, which was in line with the expression of *RIN* observed in the RT-qPCR (Figs. 5C and 4A). As *RIN* is the major regulator of system 2 ethylene (Ito et al. 2017; Li et al. 2020), this upregulation subsequently led to the increased expression of *ACS2* and *ACS4* (Fig. 2, B and C), and eventually to the ripening ethylene production. The higher ethylene production in some fruits (Fig. 2A); higher expression of *ACS2*, *ACS4*, and *RIN* (Figs. 2, B, C and 4A); and lower firmness at the MG stage of *nor-cr1* fruits (Supplementary Fig. S4) demonstrate that these ripening phenomena preceded the color change in this mutant. Additionally, we observed no increase of *NAC4* expression, which further supports the severely inhibited ethylene production in the double mutant (Fig. 5G). Taken together, the downregulation of the ripening TFs in addition to ethylene biosynthetic genes might explain the difference between the delayed ripening in *nl1-cr1* and no ripening in *nor-cr1 nl1-cr1* mutants.

### *RIN* and *NOR* regulate most of the ripening progression

Both RNA-seq and RT-qPCR show that *RIN* is significantly downregulated in the single and double mutants of *nor-cr1* and *nl1-cr* during ripening, suggesting that it is regulated by both *NOR* and *NL1*. While the role of *RIN* in ripening regulation is known (Ito et al. 2017; Li et al. 2020), it is also relevant to know its contribution relative to *NOR* and *NL1* and its position in the ripening network. To investigate this further, we created the *rin-cr1* knockout mutant using CRISPR/Cas9 and compared its phenotype and gene expression to that of the other mutants. The *rin-cr1* mutant exhibited a 22 bp deletion in the third exon of *RIN*, which resulted in a 104 aa truncated protein that aligns with the wild-type *RIN* product for the first 93 residues, comparable to the *rinG1* mutant (Ito et al. 2015) (Supplementary Fig. S1C). We also crossed *rin-cr1* with both *nor-cr* mutants and segregated the homozygous double mutants to study the combined roles of *RIN* and *NOR* in ripening regulation.

**Figure 6.**
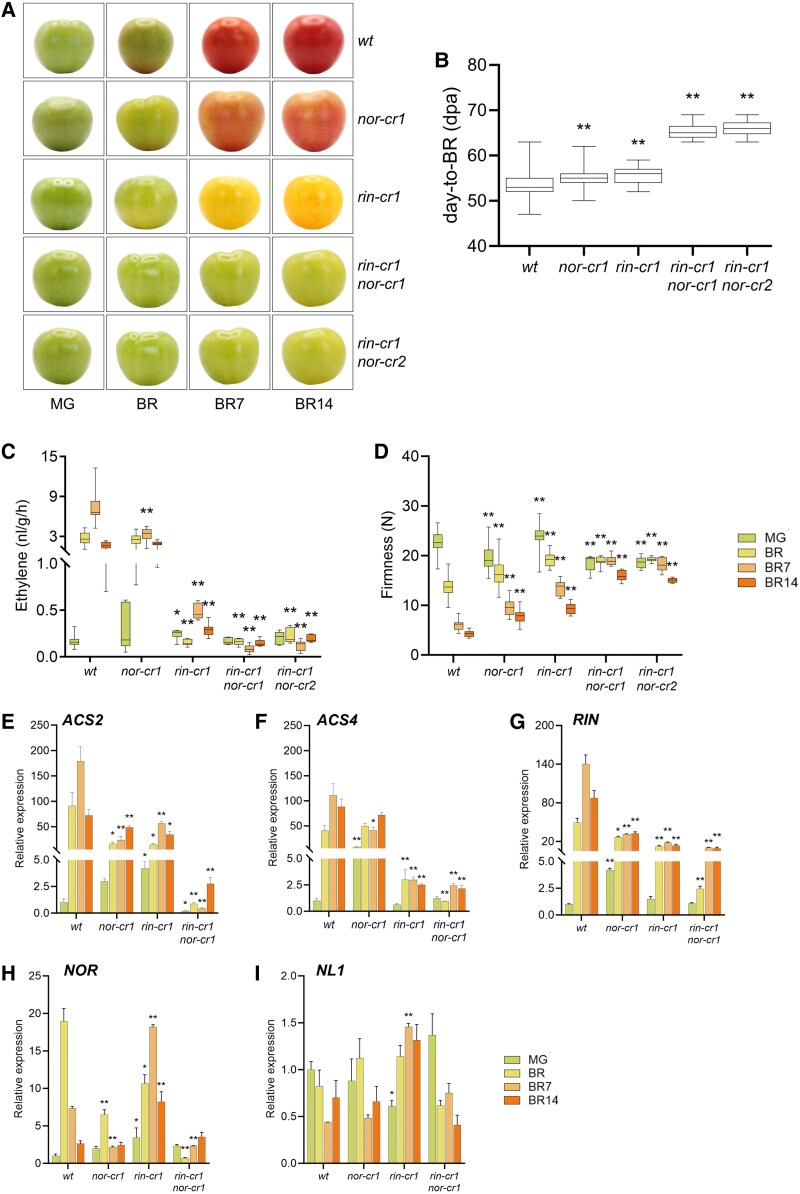
Fruit ripening phenotypes and gene expression in *nor-cr1*, *rin-cr1*, and 2 *rin-cr1 nor-cr* double homozygous mutants. **A)** Fruit appearance, **B)** days-to-breaker; **C)** ethylene production; and **D)** fruit firmness. At least 5 fruits per stage from each genotype were used for the ethylene and firmness measurements, respectively. For comparison of fruit appearance in panel (**A**), the fruit of the wild type and *nor-cr1* from Fig. 1B were reused. For the box plot in panels (**B**) to (**D**), the center line represents the median; box limits indicate the upper and lower quartiles (Q3 and Q1, respectively); and whiskers extend to 1.5 times the interquartile range (IQR) from the quartiles. Gene expression measurement using RT-qPCR of **E)**  *ACS2*; **F)**  *ACS4*; **G)**  *RIN*; **H)**  *NOR*; and **I)**  *NL1*. For the bar graphs in panels (**E**) to (**I**), error bars represent the standard error (SE) of the mean, with sample sizes of *n* = 3 biological replicates for each treatment. Asterisks indicate statistical significance based on a 2-tailed Student's *t*-test at *P* < 0.05 (*) and *P* < 0.01 (**) between mutants and wild type at the same developmental stage.

The *rin-cr1* mutant developed yellow-colored fruits after the breaker stage that turned to slightly orange at the breaker + 14 d stage (Fig. 6A), which was similar to the phenotype observed in the *rin* CRISPR mutants from previous studies (Ito et al. 2017, 2015). The fruits of *rin-cr1* were strikingly different from *nor-cr1* fruits, which developed a more orange-red color, confirming that the knockout of *RIN* results in more severe effects on ripening (Fig. 6A). When both mutations were combined, the resulting double mutants *rin-cr1 nor-cr1* and *rin-cr1 nor-cr2* changed color much slower than both single mutants, giving a pale-yellow color at the breaker + 14d stage (Fig. 6A). Although both *rin-cr1* and *nor-cr1* single mutants required a few more days to reach breaker (55 dpa, from 53 in the wild type), their combined effects of double homozygous mutations delayed it much further (to 65 dpa) (Fig. 6B).

The *rin-cr1* mutant produced substantially less ethylene than both wild type and the *nor-cr1* mutant (Fig. 6C), indicating that *RIN* plays the dominant role in regulating ethylene production during ripening progression, which is in line with its more severe color phenotype. Although ethylene production was low in the *rin-cr1* mutant at the breaker stage, we observed a slight increase over time, suggesting that while *RIN* has the dominant role, it coregulates ethylene production with other factors. Interestingly, we observed even lower ethylene levels with no further increase over time in both *rin-cr1 nor-cr* double mutants (Fig. 6C). This indicates that the residual rise in ethylene production in the *rin-cr1* mutant might be due to *NOR*, showing that ripening-related ethylene production is coregulated by *RIN* and, to a much lesser extent and independent of *RIN*, by *NOR*. To see whether ethylene could induce and accelerate ripening in these mutants, we treated the fruits with ethephon solution and observed the color change up to 14 d afterward (Supplementary Fig. S7). The treated fruit of the *rin-cr1* mutant ripened earlier and accumulated more color than the mock-treated fruit, indicating that the ethylene treatment could still progress ripening. In contrast, the mock- or ethephon-treated fruits of both double mutants initiated ripening with a similar delay as the color change shows, indicating that ethylene could not induce ripening in the absence of *NOR*.

In addition to ethylene production, we also compared fruit softening during ripening. As shown in Fig. 6D, both *nor-cr1* and *rin-cr1* single mutants softened albeit slower than the wild type. Softening was significantly reduced in the double mutants as the fruit firmness did not decrease from the MG to breaker + 7 d stage and only slightly so at breaker + 14 d. Thus, although the regulation of fruit softening by *RIN* and *NOR* may well be through ethylene signaling, the slight difference in softening rate observed between these mutants does not reflect their large difference in ethylene production.

A comparison of system 2 ethylene biosynthesis gene expression (*ACS2* and *ACS4*) revealed differences between the single and double mutants. Although both single mutants had lower *ACS2* expression compared to the wild type, the expression in the *nor-cr1* was significantly lower than in *rin-cr1* at breaker + 7 d (Fig. 6E; Supplementary Table S2). This was in contrast to *ACS4* expression, which was downregulated more in *rin-cr1* than in *nor-cr1* (Fig. 6F). Moreover, the *rin-cr1 nor-cr1* double mutant expressed *ACS2* even lower than that of both single mutants but expressed *ACS4* like the single *rin-cr1* mutant (Supplementary Table S2). The latter shows that *NOR and RIN* additively regulate *ACS2*, while *ACS4* is regulated primarily by *RIN*. The differential regulation of *ACS2* and *ACS4* also suggests why *rin-cr1* had more impact on ethylene production than *nor-cr1*.

Other ripening genes like those acting in carotenoid biosynthesis (*GGPPS2* and *PSY1*) and cell wall metabolism (*PG2a* and *PL*) were affected similarly to ethylene biosynthesis. The expression of these genes was downregulated in the single mutants and even further in the double mutant (Supplementary Fig. S8, A to D; Supplementary Table S2). Interestingly, the expression of *GGPPS2*, *PG2a*, and *PL* was more downregulated in *nor-cr1* than in *rin-cr1* at the breaker + 7 d stage, confirming that they are also directly regulated by *NOR* as previously shown (Gao et al. 2020), in addition to being regulated by ethylene.

To see whether the phenotype is correlated with the expression of ripening TF genes, we also compared the expression of *RIN*, *NOR*, and *NL1* between the wild type and the mutants. The expression of *RIN* was downregulated in both *nor-cr1* and *rin-cr1* single mutants, indicating that its expression is indirectly regulated by ethylene or directly via transcriptional regulation by NOR and RIN (Fig. 6G). The expression of *NOR* was downregulated in the *nor-cr1* knockout, while it was only shifted in the *rin-cr1* single mutant (Fig. 6H). This was in contrast with the expression of *NL1*, which was hardly affected in all mutants (Fig. 6I).

## Discussion

### Ripening initiation is mainly regulated by *NL1*

Once initiated, climacteric ripening is an irreversible process that changes the physiology and biochemistry of the fruit; with for tomato, the most common changes are ethylene production, chlorophyll degradation, carotenoid accumulation, and softening, preceded by a respiratory peak. Ethylene plays a central role in climacteric ripening as it is required to trigger the ripening process (Kidd and West 1945; Pratt and Goeschl 1969). The mechanism of ripening initiation, which is concurrent with the transition from the autoinhibitory system 1 to the autocatalytic system 2 ethylene biosynthesis, is not entirely understood. Initially, 2 hypotheses were proposed to explain this transition: the accumulation of endogenous ethylene (Klee 2004) and the change in fruit sensitivity to ethylene (Kevany et al. 2007). The autoinhibitory nature of system 1 prevents it from accumulating ethylene; therefore, it is unlikely to be responsible for initiating ripening. However, perturbation of *ACS2* and *ACS4* expression that delayed ripening initiation (Oeller et al. 1991; Hoogstrate et al. 2014) suggests that system 2 ethylene has been activated before ripening and is responsible for the transition to ripening. On the other hand, early ripening initiation could also be achieved by increasing ethylene signaling via silencing ethylene receptor genes (*NR* and *ETR4*) (Tieman et al. 2000; Kevany et al. 2007). Thus, both ethylene production and signaling play a crucial part in determining the timely ripening initiation in tomato.

Our study shows that the *nl1* knockout mutant significantly delays ripening initiation (Fig. 1A). This delay is caused by the decrease of ethylene production due to lower *ACS2*, *ACS4*, and *ACO6* expression before ripening (Figs. 2, B, C and 5D). This observation aligns with the proposed endogenous ethylene accumulation hypothesis (Klee 2004), implying that an ethylene threshold exists, such that ripening initiation will occur once it is reached. We propose that such a threshold is higher than what can be achieved by autoinhibitory system 1, ensuring that ripening would only initiate with additional ethylene produced by system 2. The precocious initiation of ripening by exogenous ethylene application in the *nl1-cr* mutants (Fig. 3) and the capacity of *NL1* to directly activate both *ACS2* and *ACS4* (Gao et al. 2018) further support that *NL1* initiates ripening by activating system 2 ethylene biosynthesis genes.

In addition to ethylene biosynthesis, ripening initiation in tomato is also marked by global hypomethylation, particularly in the promoters of ripening genes, which is facilitated by the cytosine-demethylase DML2 (Zhong et al. 2013; Liu et al. 2015). It was later shown that the DML2 transcript needs to be demethylated by an RNA m6A demethylase, ALKBH2, to ensure successful translation (Zhou et al. 2019). In line with this, our study also shows that both *DML2* and *ALKBH2* are downregulated in the *nl1* knockout mutant before ripening (Fig. 5, E and F), and this would contribute to the ripening delay in this mutant. Although there is no evidence for the direct activation of *DML2* and *ALKBH2* by *NL1* to date, it has been shown that *NOR* can bind to the promoter and activate *DML2* expression, which in turn demethylates the promoter of ripening genes, including *NOR*, to increase their expression rates in a positive feedback cycle (Gao et al. 2022). Considering the high protein sequence similarity between NL1 and NOR, their shared DNA binding site motif, and the fact that *NL1* is expressed before *NOR*, we hypothesize that NL1 first initiates this cycle, which NOR then continues. This hypothesis is supported by the findings of similar ripening-related demethylation in peach by *PpDML1*, which is regulated by *PpNAC1*, the putative ortholog of tomato *NOR* (Cao et al. 2023; Zhang et al. 2023).

Summarizing, we conclude that the downregulation of system 2 ethylene biosynthesis and of demethylation-promoting genes before ripening is the main cause of delayed ripening initiation in *nl1* mutants. However, the fact that ripening still occurs shows that ripening initiation is not regulated by *NL1* only. In addition to *nl1* mutants, it was shown that in *NAC4*-RNAi lines, ripening initiation is also delayed, although not as much as in the *nl1* mutant (Zhu et al. 2014). Since *NAC4*, like *NL1*, is also expressed from early fruit development onwards, it is a good candidate for maintaining ripening initiation in the absence of *NL1*. This is also supported by its downregulation in the *nor-cr1 nl1-cr1* double homozygous mutant, which fails to initiate ripening completely.

### *RIN*, *NOR*, and *NL1*, in decreasing order, maintain the ethylene positive feedback loop for ripening progression

Following initiation, ripening progression relies on continuous ethylene production in order to maintain the active expression of downstream ripening genes. Ethylene production must be positively regulated, which sets apart the autocatalytic system 2 from the autoinhibitory system 1 (Barry et al. 2000). Several ripening TFs, including the widely known (RIN, NOR, FUL1) and the recently added (NL1, NAC4), participate in regulating system 2 ethylene, hence placing them upstream of the ethylene pathway. The mild ripening phenotypes in the true knockout (as opposed to spontaneous) mutants of *rin* and *nor* clearly show that ripening progression, in terms of ethylene production, is extensively regulated by multiple ripening TFs. This raises the question whether these TF-encoding genes act epistatically or independently to regulate ethylene production.

In this study, we sought to clarify the relative contributions of *NOR* and *NL1*, 2 highly similar NAC TF genes, and of *RIN* in regulating tomato fruit ripening. In accordance with the previous studies (Gao et al. 2018, 2020; Wang et al. 2019b), the *nl1* mutants delay ripening initiation, and both *nl1* and *nor* mutants inhibit ripening progression. We demonstrated that the ripening phenotypes (i.e. ethylene production, color development, and fruit softening) and corresponding gene expression decrease gradually as more functional alleles of *NOR* or *NL1* are removed. Moreover, the inability to induce ripening by exogenous ethylene when both *NOR* and *NL1* are knocked out (Fig. 3) suggests that at least 1 functional gene is required for ripening to progress and that they control more than only the ethylene biosynthesis pathway. As mentioned earlier, ripening also requires a developmental trigger in the form of cytosine demethylation by DML2 (Liu et al. 2015; Lang et al. 2017). Although ethephon treatment ultimately compensates for the NL1 deficiency, we hypothesize that in addition to NL1, active NOR is required for expression of *DML2* and that without this, RIN will not be induced. This is corroborated by the observation that, in *dml2* mutants, ectopic, methylation-independent expression of *RIN* compensates for a large part of the ripening defects (Niu et al. 2025). It is further corroborated by the RNA-seq and RT-qPCR data (Figs. 4A to D and 5D to G), showing that most of the ripening genes are severely downregulated in the *nor nl1* double mutant. Among these downregulated genes are *RIN*, *NAC4*, and *FUL1*, which we hypothesize to be downstream of both *NOR* and *NL1*. Both RIN and FUL1 belong to the MADS domain TF family and regulate ripening. They have been shown to interact with each other at the protein level (Fujisawa et al. 2014), and their knockout significantly reduces ethylene production and the expression of system 2 ethylene genes (Ito et al. 2017; Wang et al. 2019b; Li et al. 2020; Jia et al. 2024). The pivotal role of *RIN* is apparent in its knockout mutant, which greatly downregulates ethylene production and consequently inhibits ripening progression. However, the presence of functional *NOR* still enables it to slowly produce ethylene, contributing to the slight color change later on. Knocking out both *RIN* and *NOR* inhibits ethylene production and consequently ripening progression even further, as shown by much slower color development and the inability to progress faster with exogenous ethylene (Supplementary Fig. S7). However, the relatively higher expression of *ACS2* and *ACS4* in *rin nor* than in *nor nl1* double mutants (Supplementary Table S2) explains that the former underwent minor changes related to ripening later on, attributable to the residual *NL1* activity.

While *RIN*, *NOR*, and *NL1* regulate ripening progression via ethylene production, their expression increases, coinciding with ethylene production, suggesting that they themselves are regulated by ethylene signaling. This is supported by the direct activation of *RIN*, *NOR*, and *FUL1* expression by *EIL1* (Huang et al. 2022). Together, this shows that the expression of major ripening TFs, like *RIN*, *NOR*, and *FUL1*, is regulated by the ethylene signaling pathway that forms a positive feedback loop, defining the autocatalytic nature of system 2. In addition to being regulated through ethylene signaling, these TFs likely regulate each other's expression directly. ChIP-chip data demonstrated that *RIN* directly bound the *NOR* promoter (Fujisawa et al. 2013, 2012). Other ChIP-seq data show the presence of NAC binding sites in the promoter of *RIN*, indicating that NOR could also directly activate *RIN* expression (Gao et al. 2022). In this study, we showed that *RIN* expression was downregulated in the *nor* knockout mutants (Fig. 4A), while *NOR* expression remained unchanged in the *rin* knockout mutant (Fig. 6H), making *NOR* an upstream regulator of *RIN*. *NL1* possibly controls the expression of *NOR*, since the same ChIP-seq data showed the presence of an NAC binding site in the *NOR* promoter, suggesting that NAC TFs regulate it in addition to EILs. Considering that the expression of *NOR* was significantly lower at the MG stage in *nl1* knockout mutants (Fig. 4B), and *NL1* expression precedes the expression of *NOR*, we hypothesize that NL1 binds to the *NOR* promoter at this NAC binding site to activate its expression at the ripening initiation. We did not find either NAC or EIN3 binding sites in the promoter of *NL1* (Lü et al. 2018; Gao et al. 2022), suggesting that although *NL1* controls ripening by activating ethylene biosynthesis genes, it is not a part of the positive feedback loop.

### The ripening regulatory model

**Figure 7.**
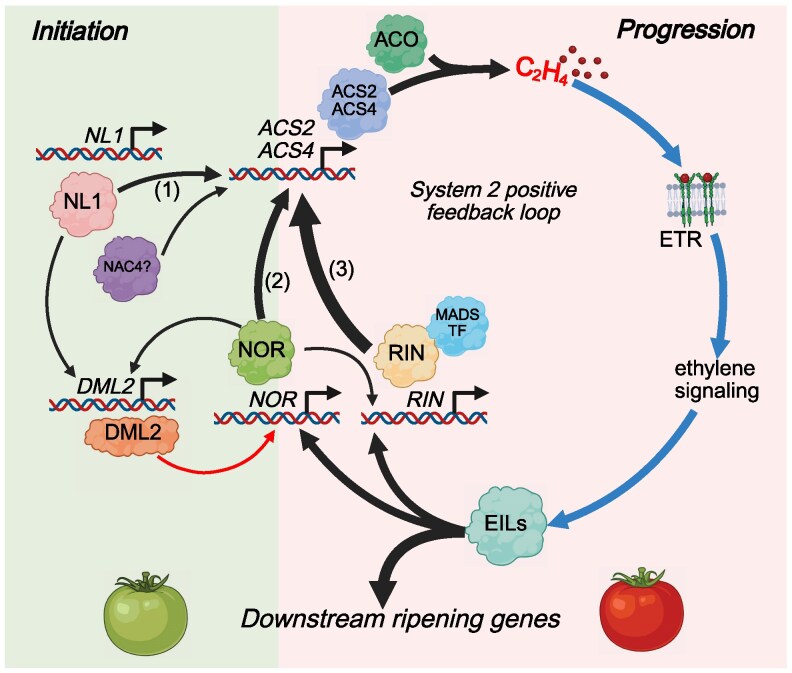
Model of ripening regulation by NL1, NOR, and RIN. Arrow thickness and color indicate the strength and type of regulation (black = transcriptional regulation, red = promoter demethylation, blue = ethylene signal transduction.) The order of system 2 ethylene activation proceeds from (1), (2), to (3). While promoter demethylation by DML2 affects many ripening genes, it is only depicted towards *NOR* in this model to avoid complexity.

We have shown in this study that *NOR* and *NL1* together affect the entire tomato ripening process via the regulation of ethylene production, ripening demethylase DML2, and expression of other ripening TFs, mainly RIN. We propose a model of ripening regulation by NOR and NL1 as shown in Fig. 7. In this model, ripening is initiated by the activation of system 2 ethylene production, as a result of *ACS2* and *ACS4* expression being activated by NL1 and possibly NAC4 (Zhu et al. 2014). Simultaneously, NL1 activates the expression of *DML2*, which demethylates the promoter regions of ripening-related genes, particularly of *NOR*, making them accessible for transcriptional activation. Similar to NL1, NOR protein can also activate *DML2* expression further, establishing a positive feedback loop that ensures continuous demethylation of the *NOR* promoter and sustains the expression of *DML2* (Gao et al. 2022). The produced ethylene activates the ethylene signaling pathway, leading to the involvement of EILs, which activate the expression of ripening TF genes such as *NOR* and *RIN* (Lü et al. 2018; Huang et al. 2022). Both NOR and RIN, in turn, further activate the expression of *ACS2* and *ACS4* to produce more ethylene(Ito et al. 2017; Gao et al. 2020), creating a positive feedback loop that maintains ethylene production required for ripening progression.

In the *nor* mutant, the ripening progression is inhibited by attenuation of the system 2 loop, resulting in lower ethylene production (Supplementary Fig. S9A). This contrasts with the *nl1* mutant, which only delays the activation of the system 2 loop due to slower ethylene accumulation. When enough ethylene is supplied to activate the loop, ripening progresses similarly to normal (Supplementary Fig. S9B). Like *nor*, the *rin* mutant inhibits ripening progression by decreasing the ethylene output of the system 2 loop but with a more significant effect (Supplementary Fig. S9C). The combination of *nor* and *rin* mutations interrupts the loop and makes it unable to ramp up production by ethylene signaling. However, a small amount of ethylene could still be produced via *NL1*-activated system 2 genes, causing a slight color change indicative of ripening (Supplementary Fig. S9D). Finally, the combination of *nor* and *nl1* mutations abolishes ripening through the absence of the system 2 loop and the nonloop ethylene biosynthesis (Supplementary Fig. S9E). Therefore, we conclude that not a single gene, but the NOR/NL1 module is the master regulator of tomato ripening.

## Materials and methods

### Plant materials

Tomato (*S. lycopersicum*) cv. Moneyberg (wild type) and cv. Ailsa Craig (wild type and *nor*) were used. All mutant lines used in this study were generated either by *Agrobacterium*-mediated transformation or by crossings between the single mutants. Lines containing *nl1* mutations (*-cr1* and *-cr2*) were generated in this study, while the *nor* (*-cr1* and *-cr2*) and *rin* (*rin-cr1*) mutants were produced from earlier studies (Wang et al. 2019b). The *nor nl1* double mutant was obtained by crossing the *nor-cr1* with *nl1-cr1*, while the *rin nor* double mutants were obtained by crossing the *rin-cr1* with *nor-cr1* and *nor-cr2*. Seeds were germinated in the dark, and seedlings were placed in a growth chamber at 25 °C and 65% humidity with a 16 h light and 8 h dark cycle until 25 cm height and subsequently in the greenhouse facility of Unifarm, Wageningen University & Research. Phenotyping and seed collection were conducted at the greenhouse. For plant phenotyping and gene expression analysis, we used the segregated progeny of T_0_ lines without the transgenic mutagenic insert. Phenotyping was conducted on fruits of 3 plants at 4 developmental stages, namely, 30 d postanthesis (30 dpa), mature green (MG), breaker (BR), breaker + 7 d (BR7), and breaker + 14 d (BR14). Details of phenotyping parameters are described in the following subsections. Three biological replicates from each developmental stage were used for gene expression analysis using RT-qPCR, where a pool of 3 fruits from the same plant constitutes 1 biological replicate.

### CRISPR/Cas12a mutagenesis, tomato transformation, and genotyping

Guide design for the CRISPR/Cas12a system was conducted using the CRISPOR webserver (Haeussler et al. 2016; Concordet and Haeussler 2018). We selected 2 guides targeting the first exon of *NL1*. A binary level 2 vector containing expression cassettes was constructed using Golden Gate cloning and parts from the MoClo toolkit, a gift from Sylvestre Marillonnet (Addgene kit #1000000044) and the MoClo plant parts kit, a gift from Nicola Patron (Addgene kit #1000000047) (Weber et al. 2011; Werner et al. 2012; Engler et al. 2014). A level 1 vector for sgRNA cloning behind an *Arabidopsis thaliana* U6-26 promoter was a gift from Mark Youles (The Sainsbury Laboratory). This vector includes an operon that expresses a red fluorescent protein (RFP), which can be replaced with a spacer via *Bsm*BI restriction sites and allowing pink/white colony selection to identify successful sgRNA integration. All necessary components in level 1 vectors, comprising (i) *pICH47732::pNOS-NPTII-tOCS*, (ii) *pICH47742::pPcUbi4-2xintron-ttLbCas12a-tNOS*, (iii) *pICH47751::2xproCaMV35S-tGFP-tCaMV35S*, (iv) *pICH47761::pAtU6_26-(BsmB1)-Cpf1PinkLbShortRepeat* containing gRNA1, (v) *pICH54055* dummy, (vi) *pICH47781::pAtU6_26-(BsmB1)::Cpf1PinkLbShortRepeat* containing gRNA2, and (vii) *pICH41822* end-linker, were assembled to a level 2 vector (*pICSL4723*) (Weber et al. 2011; Slaman et al. 2023). The primers used for constructing crRNA vectors and for genotyping are listed in Supplementary Table S7. Transformation of cotyledon explants with *Agrobacterium tumefaciens* C58 harboring the level 2 CRISPR/Cas12a construct was conducted according to van Roekel et al. (1993).

### Day-to-BR and fruit appearance

Day-to-BR was defined as the number of days spent from flower anthesis to the breaker stage indicated by the first observation of color change at the stylar end of the fruit. Fruit pictures were taken using a DSLR camera with a white background.

### On-the-vine whole fruit firmness

On-the-vine whole fruit firmness was measured with the Fruit Texture Analyser (FTA) GS-15 (Güss, South Africa) as the average of 4 measurements along the equator of each fruit, of the amount of force (*N*) needed for a 1 cm diameter probe to make a 2 mm depression.

### Ethylene production

Harvested fruit was left in the open air for 1 h to release any wound-induced ethylene production. Subsequently, the fruit was incubated in a closed jar for 2 h, and then a 1 ml air sample from the jar's headspace was injected into TRACE 1300 gas chromatograph (Thermo Fisher Scientific, United States) coupled with a flame ionization detector (FID, temperature = 260 °C, cycle time = 3 min) using an Agilent J&W GS-GasPro 10 m 0.32 mm ID column coupled with an Agilent J&W GS-GasPro 20 m 0.32 mm ID column. The measured ethylene peak was compared with an ethylene calibration curve (98 to 970 ppb) to give the ethylene concentration. Ethylene production was defined as the volume (nl) of ethylene produced per hour per gram fruit according to the following formula:


$$C_{2}H_{4}.g^{-1}.h^{-1}=\frac{C_{C_{2}H_{4}}(V_{j}-V_{t})}{m\times \Delta t}$$

where $C_{2}H_{4}.g^{-1}.h^{-1}$ is the volume of ethylene produced (nl) per hour per gram fruit weight; $C_{C_{2}H_{4}}$ is measured ethylene concentration (ppb); $V_{j}$ is the volume of the jar (ml); $V_{t}$ is volume of the fruit (ml) derived from its weight by assuming 1 g/l fruit density; *m* is the fruit weight (g); and Δt is the incubation time (h).

### Ethephon treatment

Fruits from each genotype were submerged in distilled water (mock) or 0.1% (3.32 mm) ethephon solution for 30 min. The fruits were then dried and left in the open air for further observation until 14 d. Both mock and ethephon treatments were repeated every 3 d to supplement the lacking ethylene production in the mutants. Pictures of the fruits were taken at 0, 4, 7, 10, and 14 dpt.

### Chlorophyll degradation and lycopene production

Chlorophyll degradation and lycopene accumulation were measured via remittance spectroscopy using Pigment Analyzer PA1101 (CP, Germany). The measured indexes of normalized difference vegetation index (NDVI) and normalized anthocyanin index (NAI) were used to represent both chlorophyll and lycopene content, respectively (Zude 2003; Farneti et al. 2012; Schouten et al. 2014). Five fruits from each line were measured from the breaker or 80 dpa to 7 d afterwards on a daily basis, and an average from 3-point measurements at the fruit equatorial region was taken as the representative values of both chlorophyll and lycopene.

### RNA isolation, RT-qPCR, and RNA-seq

Total RNA was isolated using the cetyltrimethylammonium bromide (CTAB) method. Ground fruit pericarp from each sample was mixed with CTAB buffer (2% CTAB containing 1% polyvinylpyrrolidone, 2 m NaCl, 100 mm Tris, and 25 mm EDTA, and 2% β-mercaptoethanol) and incubated at 65 °C for 10 min. The water phase containing RNA was separated by adding an equal amount of chloroform and then centrifuged for 10 min. RNA was precipitated by adding LiCl to a final concentration of 2 m and incubation at −20 °C for 2 h. Precipitated RNA was isolated by centrifugation for 45 min, followed by washing with 70% ethanol and drying in a vacuum desiccator. Finally, the isolated RNA was diluted in diethylpyrocarbonate (DEPC)-treated water. Subsequently, RNA was treated with TURBO DNase (Thermo Fisher Scientific) according to the manufacturer's protocol.

For RNA-seq, the RNA samples were shipped to Novogene (United Kingdom). Library preparation was conducted using a directional poly-A mRNA enrichment. Subsequently, RNA-seq was conducted using NovaSeq X Plus paired-end 150 bp. RNA-seq analysis of generating DEGs was conducted with QIAGEN CLC Genomics Workbench 22.0 (QIAGEN, Aarhus, Denmark). For RT-qPCR, 1 µg of RNA was used for cDNA synthesis using qScript cDNA synthesis kit (Quantabio). RT-qPCR reaction was performed using iQ SYBR Green Supermix (Bio-Rad) in an iCycler iQ5 system (Bio-Rad). *Actin* was selected as the reference gene, and the relative expression (RE) levels of each gene were calculated using the 2^−ΔΔCt^ method (Livak and Schmittgen 2001). All RT-qPCR data were generated using 3 biological replicates from each developmental stage, where the expression was calculated relative to the wild type at the MG stage (set at 1). Primers used for this RT-qPCR are listed in Supplementary Table S7.

### Accession numbers

The genes used in this study and their accession numbers are as follows: *ACS2*, Solyc01g095080; *ACS4*, Solyc05g050010; *ACO1*, Solyc07g049530; *ACO6*, Solyc02g03635; *PL*, Solyc03g111690; *PG2a*, Solyc10g080210; *NOR-like1* (*NL1*), Solyc07g063420; *NAC-NOR* (*NOR*), Solyc10g006880; *NAC4*, Solyc11g017470; *MADS-RIN* (*RIN*), Solyc05g01202; *PSY1*, Solyc03g031860; *GGPPS2*, Solyc04g079960; *DML2*, Solyc10g083630; *ALKBH2*, Solyc01g104130; actin, Solyc03g078400.

## Supplementary Material

kiaf291_Supplementary_Data

## Data Availability

The data supporting the findings of this work are available in the manuscript and present in the Supporting Information files (Supplementary Figs. S1 to S9; Supplementary Tables S1 to S7). All the raw sequences are deposited to the National Center for Biotechnology Information Sequence Read Archive under the accession BioProject PRJNA1230152.

## Supplementary data

The following materials are available in the online version of this article.

**Supplementary Figure S1.** Mutation diagram of *nl1*, *nor*, and *rin* knockout mutants.

**Supplementary Figure S2.** Comparison of ripening progression from breaker to breaker + 7 d between wild type and mutants.

**Supplementary Figure S3.** Inner fruit appearance of wild type and mutants.

**Supplementary Figure S4.** Fruit firmness and expression of cell wall metabolism genes.

**Supplementary Figure S5.** Gene Ontology (GO) enrichment of the downregulated DEGs from the *nl1-cr1* mutant at the mature green (MG) stage.

**Supplementary Figure S6.** Gene Ontology (GO) enrichment of the downregulated DEGs from the *nor-cr1* mutant at the mature green (MG) stage.

**Supplementary Figure S7.** Comparison between the wild type and *rin-cr1*, *rin-cr1 nor-cr1*, and *rin-cr1 nor-cr2* fruits treated with water or ethephon from 0 to 14 d posttreatment.

**Supplementary Figure S8.** Gene expression comparison between the wild type and *nor-cr1*, *rin-cr1*, and *rin-cr1 nor-cr1* mutants by RT-qPCR for carotenoid biosynthesis and cell wall metabolism genes.

**Supplementary Figure S9.** Models of ripening regulation in the mutants of *nor*, *nl1*, *rin*, *rin nor*, and *nor nl1*.

**Supplementary Table S1.** Pairwise *t*-tests of ripening-related gene expression between the wild type, single, and double mutants of *nor-cr1* and *nl1-cr1*.

**Supplementary Table S2.** Pairwise *t*-tests of ripening-related gene expression between the wild type, single, and double mutants of *nor-cr1* and *rin-cr1*.

**Supplementary Table S3.** Comparison of differentially expressed genes (DEGs) at mature green (MG) or 80 dpa between *nor-cr1*, *nl1-cr1*, and *nor-cr1 nl1-cr1* mutants.

**Supplementary Table S4.** Gene Ontology (GO) enrichment of the downregulated DEGs from *nor-cr1 nl1-cr1* mutant at 80 dpa stage.

**Supplementary Table S5.** Gene Ontology (GO) enrichment of the downregulated DEGs from *nl1-cr1* mutant at mature green (MG) stage.

**Supplementary Table S6.** Gene Ontology (GO) enrichment of the downregulated DEGs from *nor-cr1* mutant at mature green (MG) stage.

**Supplementary Table S7.** Primers used in this study.

## Dive Curated Terms

The following phenotypic, genotypic, and functional terms are of significance to the work described in this paper:

PSY1 Gramene: Solyc03g031860
PSY1 Araport: Solyc03g031860

ACS6 Gramene: Solyc08g008100
ACS6 Araport: Solyc08g008100

EIL1 Gramene: Solyc06g073720
EIL1 Araport: Solyc06g073720

ACS2 Gramene: Solyc01g095080
ACS2 Araport: Solyc01g095080

ACS4 Gramene: Solyc05g050010
ACS4 Araport: Solyc05g050010

DML2 Gramene: Solyc10g083630
DML2 Araport: Solyc10g083630

ACO1 Gramene: Solyc07g049530
ACO1 Araport: Solyc07g049530

CEL1 Gramene: Solyc08g081620
CEL1 Araport: Solyc08g081620

ALKBH2 Gramene: Solyc01g104130
ALKBH2 Araport: Solyc01g104130

ACS1A Gramene: Solyc08g081550
ACS1A Araport: Solyc08g081550

PG2a Gramene: Solyc10g080210
PG2a Araport: Solyc10g080210

PL Gramene: Solyc03g111690
PL Araport: Solyc03g111690

NAC-NOR Gramene: Solyc10g006880
NAC-NOR Araport: Solyc10g006880

NOR-like1 Gramene: Solyc07g063420
NOR-like1 Araport: Solyc07g063420

MADS-RIN Gramene: Solyc05g012020
MADS-RIN Araport: Solyc05g012020

NAC4 Gramene: Solyc11g017470
NAC4 Araport: Solyc11g017470

GGPPS2 Gramene: Solyc04g079960
GGPPS2 Araport: Solyc04g079960

FUL1 Gramene: Solyc06g069430
FUL1 Araport: Solyc06g069430

FUL2 Gramene: Solyc03g114830
FUL2 Araport: Solyc03g114830

ACO6 Gramene: Solyc02g036350
ACO6 Araport: Solyc02g036350
