# Supplementary material for: A comprehensive model of tomato fruit ripening regulation by the transcription factors NOR-like1, NAC-NOR, and MADS-RIN
Source: Plant Physiol. 2025 Jun 30;198(3):kiaf291. doi: 10.1093/plphys/kiaf291 (PMC12305540; doi:10.1093/plphys/kiaf291)
Supplement: kiaf291_Supplementary_Data [file kiaf291_supplementary_data.zip › Supplementary Figures_6.25.pdf]

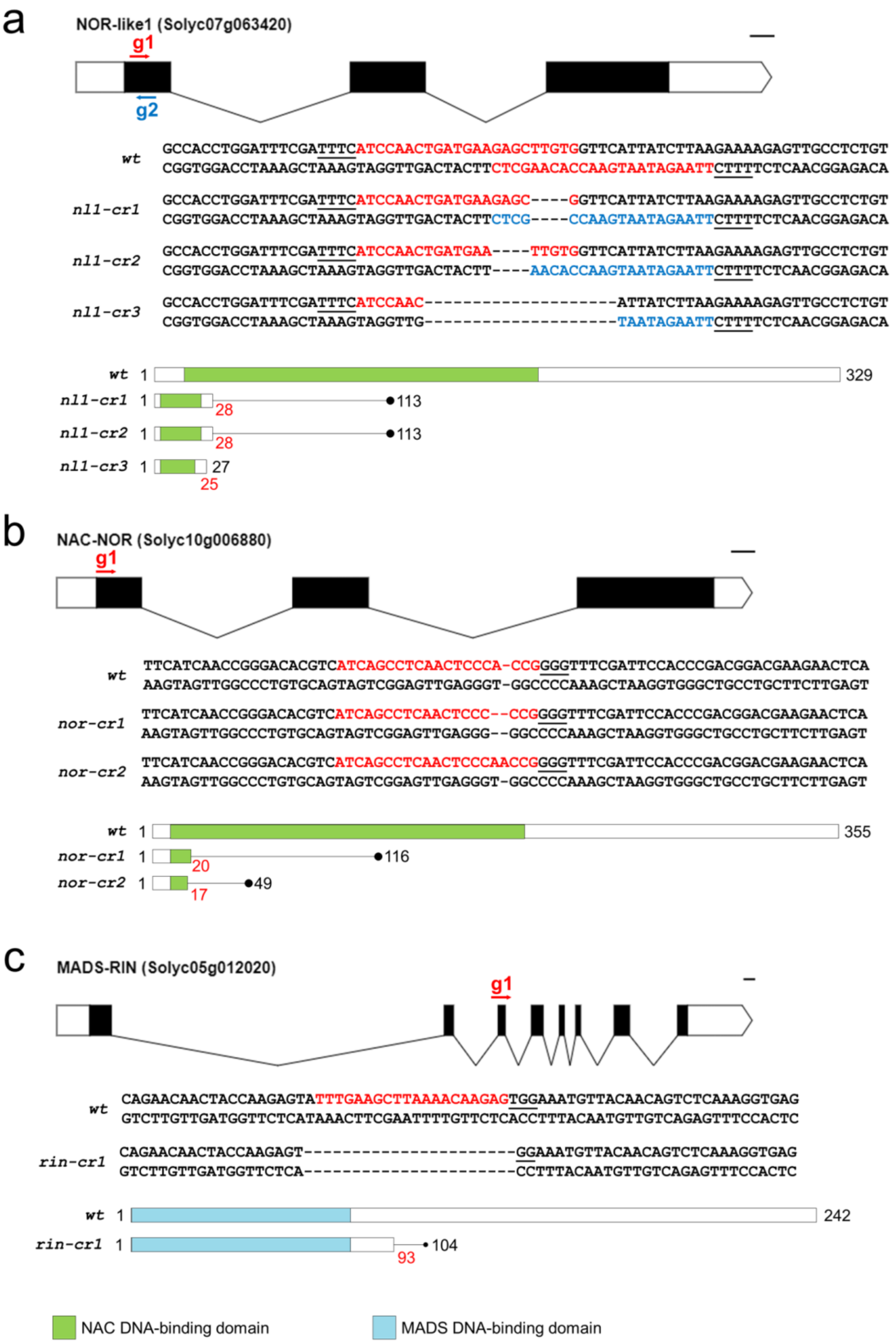

Supplementary Figure S1. Mutation diagram of (a) *nl1*, (b) *nor*, and (c) *rin* knockout mutants. Numbers in red indicates the length of amino acid in the mutant protein that aligns with the native protein

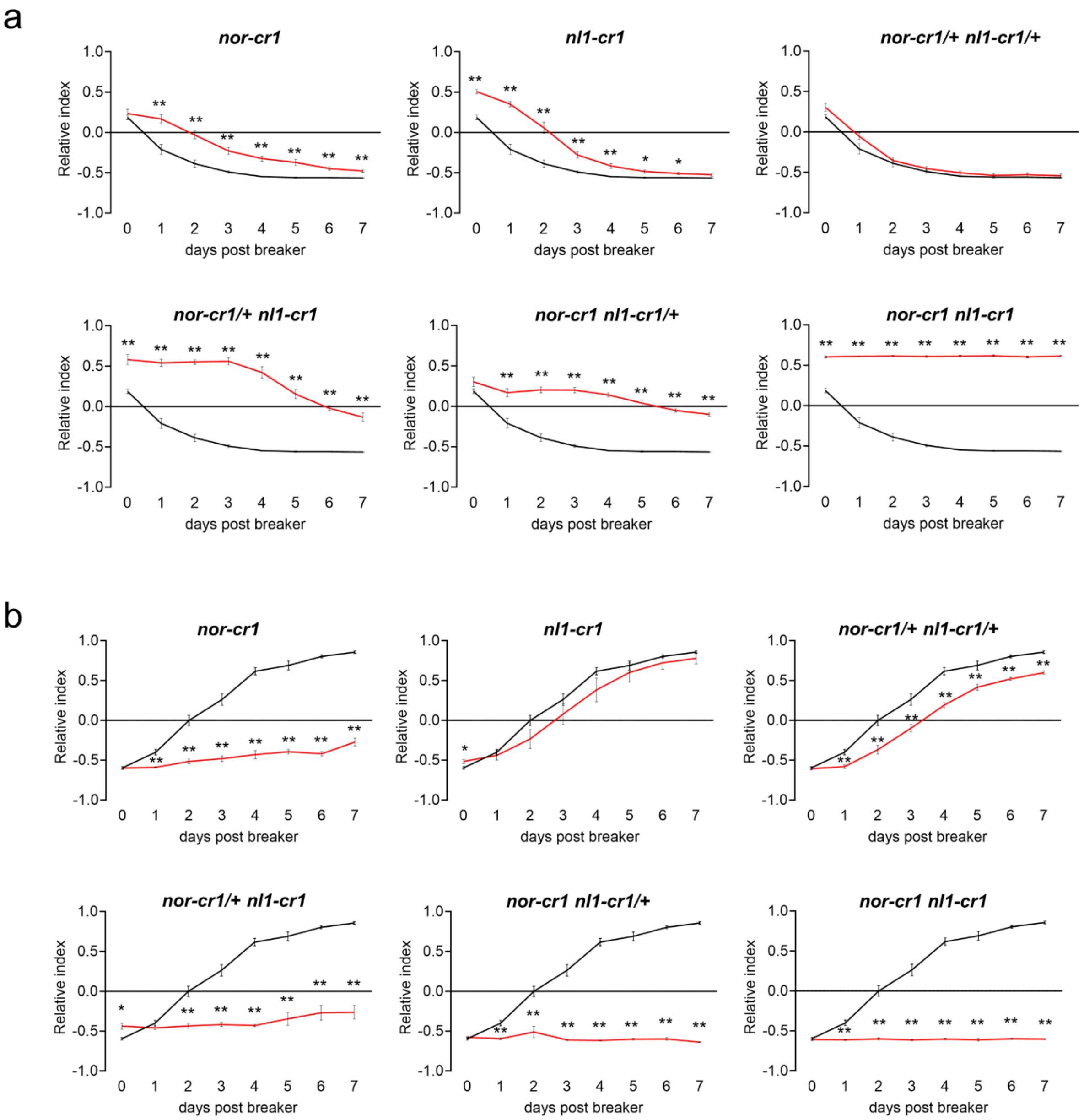

Supplementary Figure S2. Comparison of ripening progression from breaker to breaker + 7 days between wild type (black line) and mutants (red line) measured as changes of (a) chlorophyll and (b) lycopene levels. For the *nor-cr1 nl1-cr1* mutant, fruits at 80 days-post-anthesis (dpa) were used in the measurement. Asterisks indicate significant differences using a two tailed Student's *t*-test at $p < 0.05$ (*) and $p < 0.01$ (**) between the wild type and mutant.

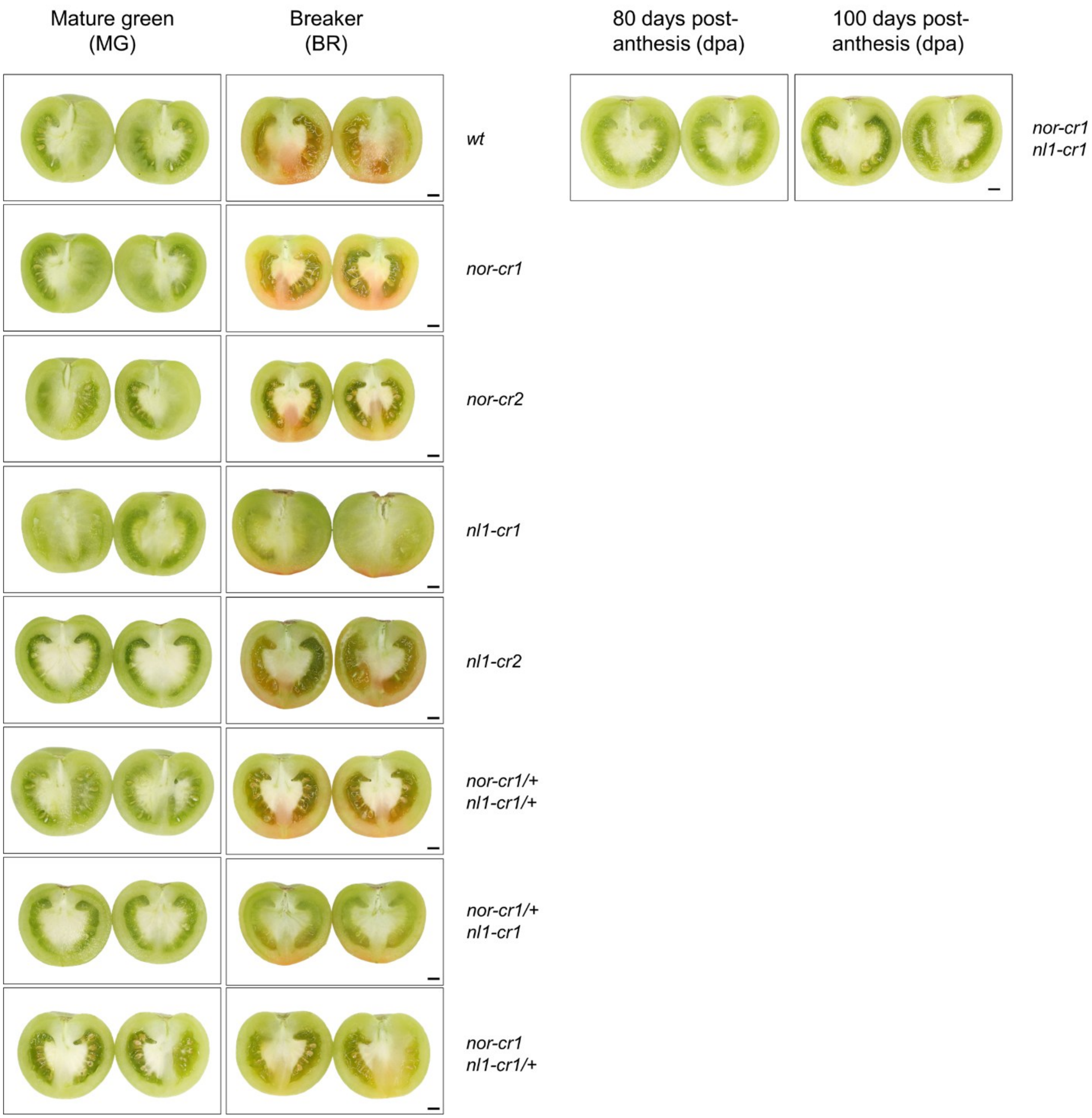

Supplementary Figure S3. Inner fruit appearance of wild type and mutants at the mature green (MG) and breaker (BR) stages. For the *nor-cr1 nl1-cr1* double homozygous mutant, fruit inner tissue appearance was taken at 80 and 100 dpa.

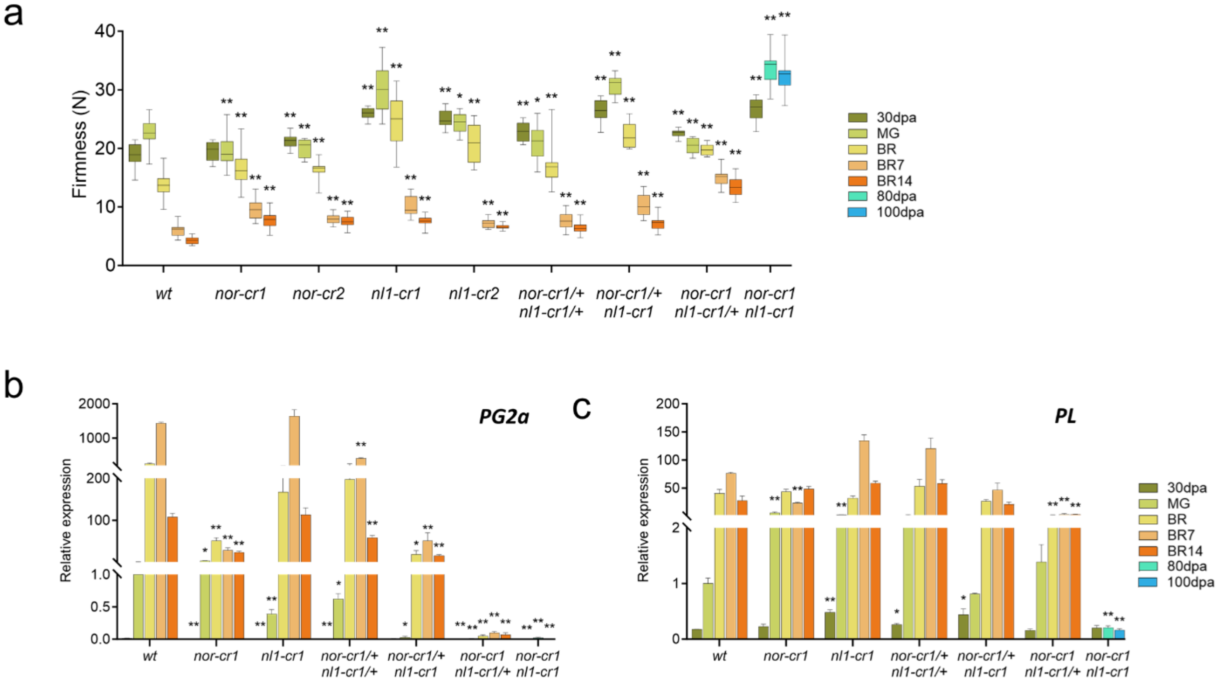

Supplementary Figure S4. Fruit firmness and expression of cell wall metabolism genes. (a) Fruit firmness of wild type and mutants. At least eight fruits per stage from each genotype were used for the measurements. Gene expression of (b) *PG2a* and (c) *PL* as measured by RT-qPCR. For the box plot in panel A, the center line represents the median; box limits indicate the upper and lower quartiles (Q3 and Q1, respectively); and whiskers extend to 1.5 times the interquartile range (IQR) from the quartiles. For the bar graphs in panels B and C, error bars indicate the standard error of the mean (SE), based on n = 3 independent experiments. Asterisks indicate significant differences using a two tailed Student's t-test at $p < 0.05$ (*) and $p < 0.01$ (**) between mutants and wild type at the same developmental stages, except for the *nor-cr1 nl1-cr1* double homozygous mutant, where both 80 and 100 dpa stages were compared to wild type mature green. Abbreviations: 30 dpa (30 days post-anthesis), MG (mature green), BR (breaker), BR7 (breaker + 7 days), BR14 (breaker + 14 days).

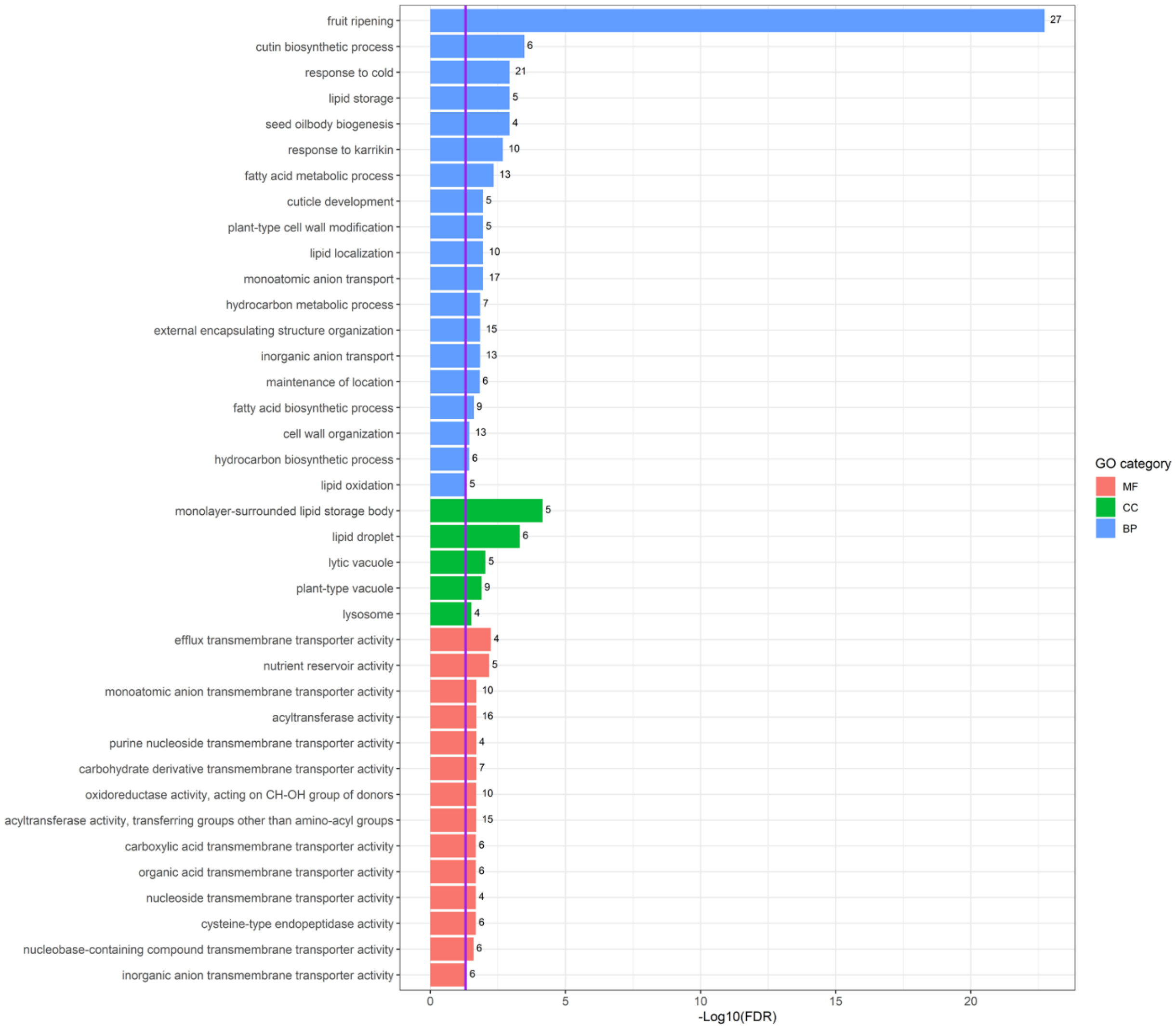

Supplementary Figure S5. Gene Ontology (GO) enrichment of the downregulated DEGs *nl1-cr1* mutant at mature green (MG) stage.

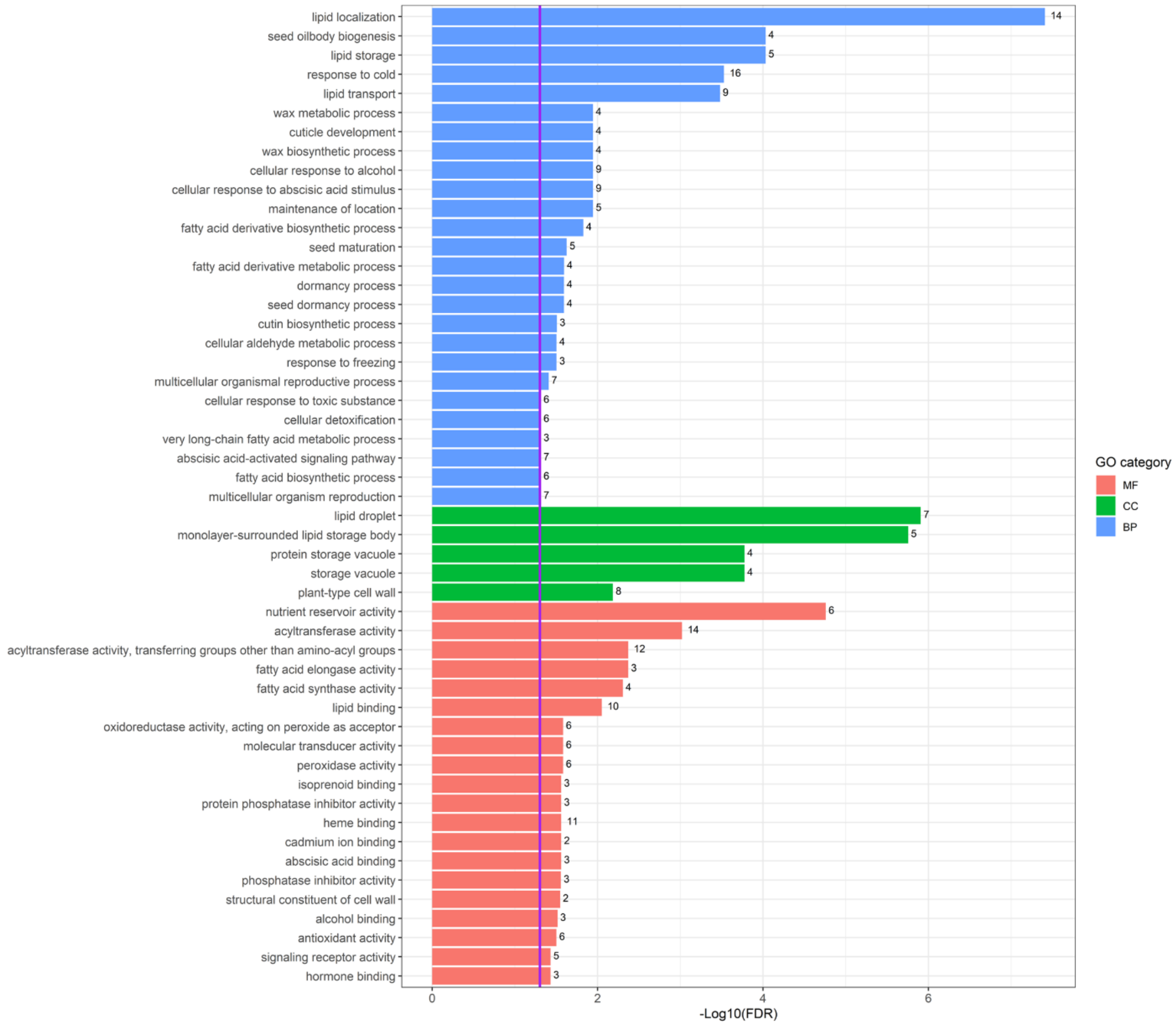

Supplementary Figure S6. Gene Ontology (GO) enrichment of the downregulated DEGs from *nor-cr1* mutant at mature green (MG) stage.

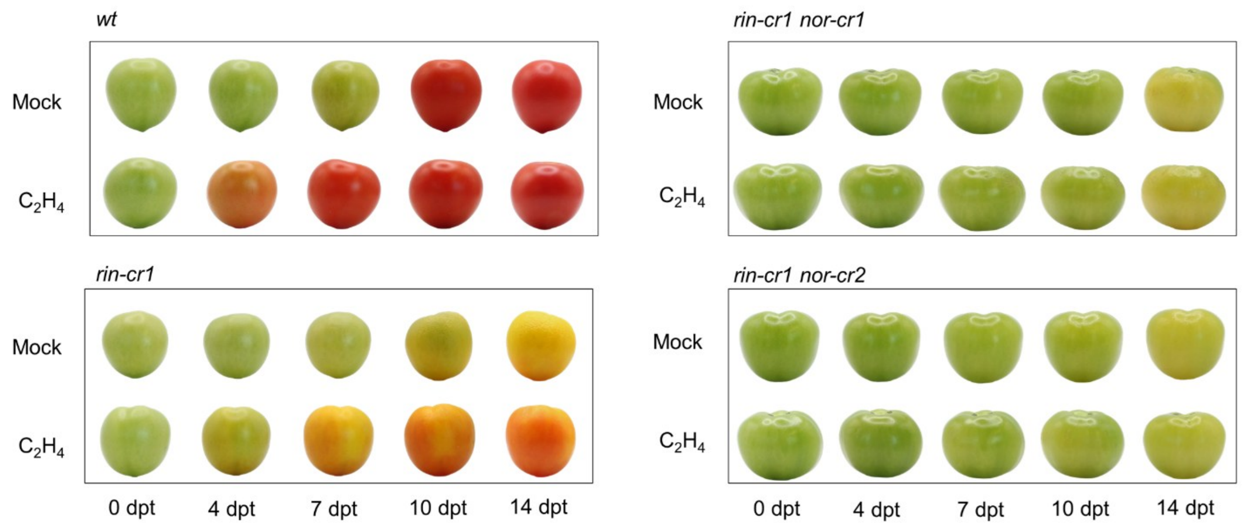

Supplementary Figure S7. Comparison between the wild type and *rin-cr1*, *rin-cr1 nor-cr1*, and *rin-cr1 nor-cr2* fruits treated with water (mock; upper rows) or ethephon (lower rows) from 0 to 14 days-post-treatment (dpt). For comparison, the mock and ethephon treatment of wild type (wt) fruit from Figure 3 were reused here. Images were digitally extracted for comparison.

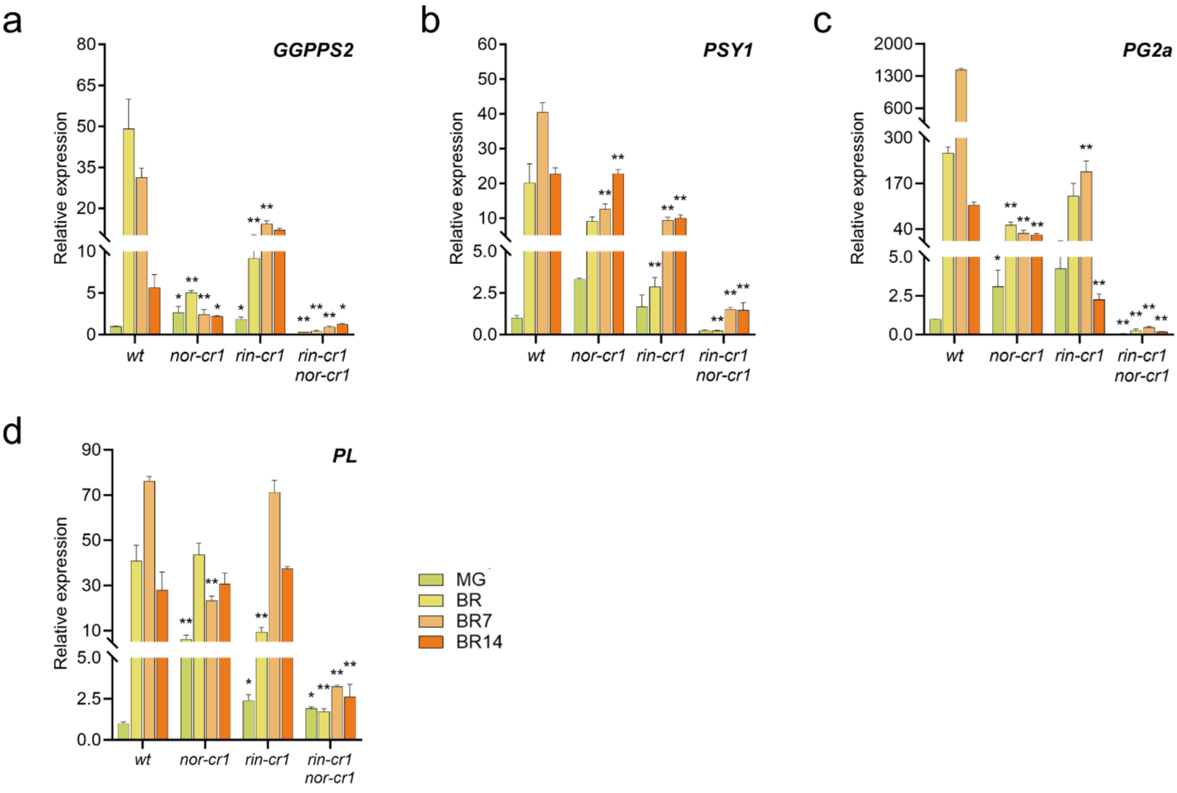

Supplementary Figure S8. Gene expression comparison between the wild type, *nor-cr1*, *rin-cr1*, and *rin-cr1 nor-cr1* by RT-qPCR for carotenoid biosynthesis pathway genes (a) *GGPPS2* and (b) *PSY1*, and cell wall metabolism genes (c) *PG2a* and (d) *PL*. For the bar graphs in all panels, error bars represent the standard error (SE) of the mean, with sample sizes of $n = 3$ biological replicates for each treatment. Asterisks indicate significant differences using a two tailed Student's *t*-test at $p < 0.05$ (*) and $p < 0.01$ (**) between each mutant and wild type at the same developmental stages.

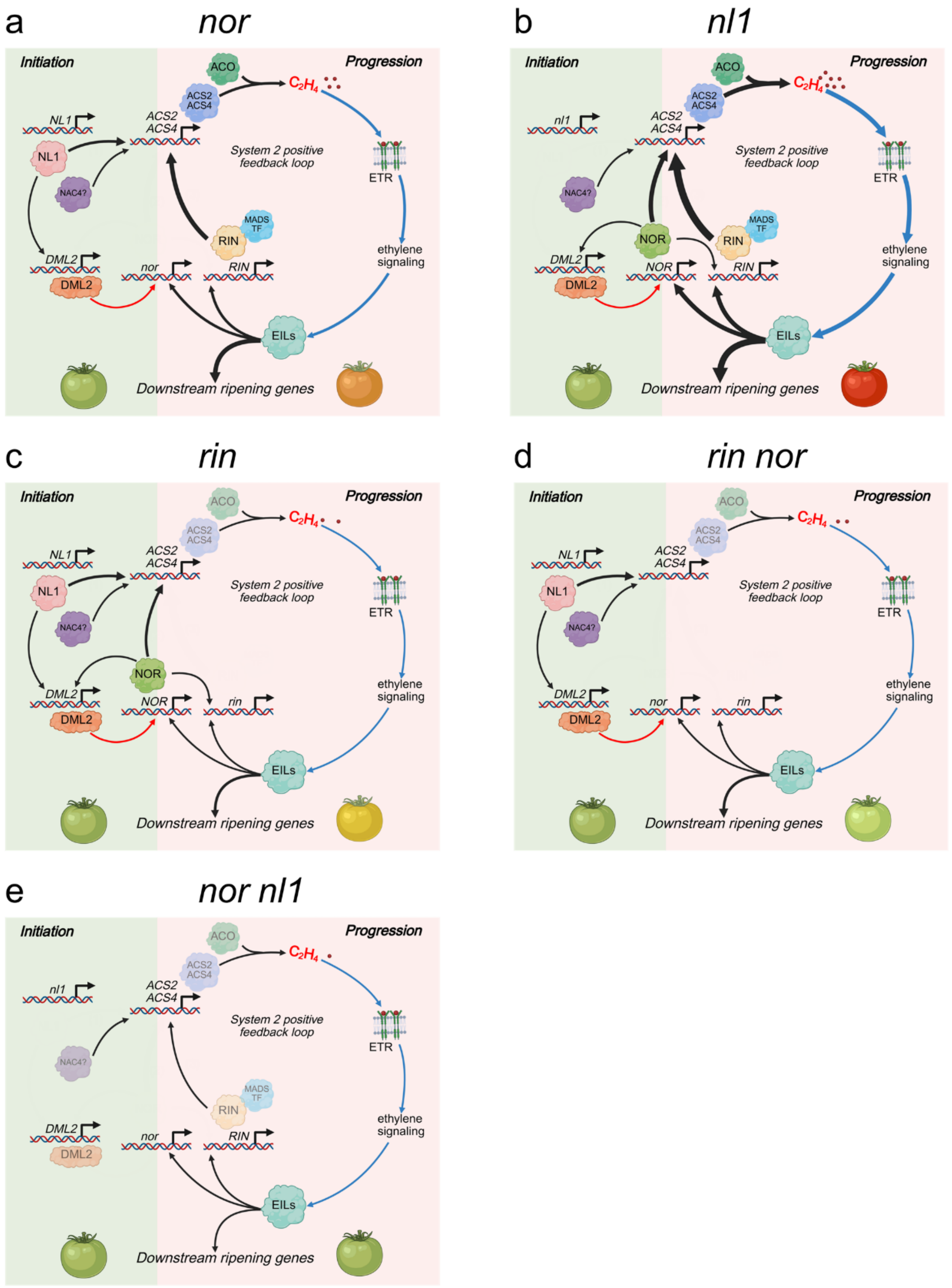

Supplementary Figure S9. Models of ripening regulation in the mutants of (a) *nor*, (b) *nl1*, (c) *rin*, (d) *rin nor*, and (e) *nor nl1*. Arrow thickness and color indicates the strength and type of regulation (black = transcriptional activation, red = promoter demethylation, blue = ethylene signal transduction.) While promoter demethylation by DML2 affects many ripening genes, it is only depicted towards *NOR* in this model to avoid complexity.
